# Apolipoprotein B-100-mediated motor neuron degeneration in sporadic amyotrophic lateral sclerosis

**DOI:** 10.1093/braincomms/fcac207

**Published:** 2022-08-22

**Authors:** Jamie K Wong, Anna K Roselle, Taylor M Shue, Serena J E Shimshak, Joseph M Beaty, Nadia M Celestin, Ivy Gao, Rose P Griffin, Merit E Cudkowicz, Saud A Sadiq

**Affiliations:** Larry G. Gluck Division of ALS Research, Tisch Multiple Sclerosis Research Center of New York, New York, NY 10019, USA; Larry G. Gluck Division of ALS Research, Tisch Multiple Sclerosis Research Center of New York, New York, NY 10019, USA; Larry G. Gluck Division of ALS Research, Tisch Multiple Sclerosis Research Center of New York, New York, NY 10019, USA; Larry G. Gluck Division of ALS Research, Tisch Multiple Sclerosis Research Center of New York, New York, NY 10019, USA; Larry G. Gluck Division of ALS Research, Tisch Multiple Sclerosis Research Center of New York, New York, NY 10019, USA; Larry G. Gluck Division of ALS Research, Tisch Multiple Sclerosis Research Center of New York, New York, NY 10019, USA; Larry G. Gluck Division of ALS Research, Tisch Multiple Sclerosis Research Center of New York, New York, NY 10019, USA; Larry G. Gluck Division of ALS Research, Tisch Multiple Sclerosis Research Center of New York, New York, NY 10019, USA; Department of Neurology, Sean M. Healey & AMG Center for ALS, Massachusetts General Hospital, Harvard Medical School, Boston, MA 02114, USA; Larry G. Gluck Division of ALS Research, Tisch Multiple Sclerosis Research Center of New York, New York, NY 10019, USA

**Keywords:** apolipoprotein B-100, sporadic amyotrophic lateral sclerosis, CSF, neurodegeneration, neurotoxicity

## Abstract

Amyotrophic lateral sclerosis is a fatal neurodegenerative disease characterized by motor neuron degeneration. Approximately 90% of cases occur sporadically with no known cause while 10% are familial cases arising from known inherited genetic mutations. *In vivo* studies have predominantly utilized transgenic models harbouring amyotrophic lateral sclerosis-associated gene mutations, which have not hitherto elucidated mechanisms underlying motor neuron death or identified therapeutic targets specific to sporadic amyotrophic lateral sclerosis. Here we provide evidence demonstrating pathogenic differences in CSF from patients with sporadic amyotrophic lateral sclerosis and familial amyotrophic lateral sclerosis patients with mutations in *SOD1, C9orf72* and *TARDBP*. Using a novel CSF-mediated animal model, we show that intrathecal delivery of sporadic amyotrophic lateral sclerosis patient-derived CSF into the cervical subarachnoid space in adult wild-type mice induces permanent motor disability which is associated with hallmark pathological features of amyotrophic lateral sclerosis including motor neuron loss, cytoplasmic TDP-43 translocation, reactive astrogliosis and microglial activation. Motor impairments are not induced by SOD1, C9orf72 or TARDBP CSF, although a moderate degree of histopathological change occurs in C9orf72 and TARDBP CSF-injected mice. By conducting a series of CSF filtration studies and global proteomic analysis of CSF, we identified apolipoprotein B-100 in sporadic amyotrophic lateral sclerosis CSF as the putative agent responsible for inducing motor disability, motor neuron degeneration and pathological translocation of TDP-43. Apolipoprotein B-100 alone is sufficient to recapitulate clinical and pathological outcomes *in vivo* and induce death of human induced pluripotent stem cell-derived motor neurons *in vitro*. Targeted removal of apolipoprotein B-100 from sporadic amyotrophic lateral sclerosis CSF via filtration or immunodepletion successfully attenuated the neurotoxic capacity of sporadic amyotrophic lateral sclerosis CSF to induce motor disability, motor neuron death, and TDP-43 translocation. This study presents apolipoprotein B-100 as a novel therapeutic target specific for the predominant sporadic form of amyotrophic lateral sclerosis and establishes proof-of-concept to support CSF pheresis as a therapeutic strategy for mitigating neurotoxicity in sporadic amyotrophic lateral sclerosis.

## Teaser

Using a novel CSF-mediated mouse model of sporadic amyotrophic lateral sclerosis, Wong et al. demonstrate that apolipoprotein B-100 induces motor disability and motor neuron degeneration. Targeted depletion of apolipoprotein B-100 in CSF via immunodepletion or CSF filtration attenuates its neurotoxic capacity, thereby preventing induction of disability and motor neuron degeneration.

## Graphical Abstract

**Graphical abstract:**
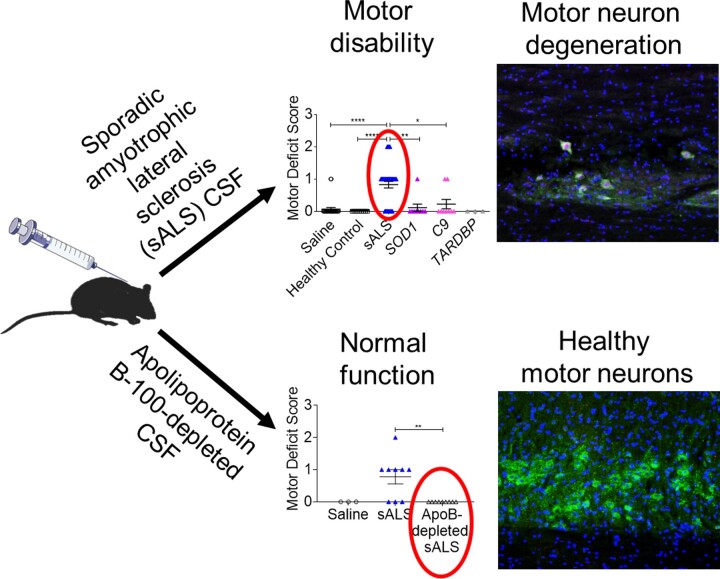

## Introduction

Amyotrophic lateral sclerosis (ALS) is a disease characterized by upper and lower motor neuron degeneration that is clinically inexorably progressive, with muscle weakness culminating in respiratory failure and death within 3–5 years.^1,2^ In ∼90% of ALS cases, the disease arises sporadically, whereas only 10% are familial cases resulting from inherited genetic mutations. Although major advancements in the ALS field have occurred with the identification of over 25 ALS-associated genes since the seminal discovery of the superoxide dismutase 1 (*SOD1*) gene in 1993,^3,4^ pathological mechanisms underlying the sporadic form of the disease remain unclear and therapeutic options are lacking. Successful disease-attenuating therapies tested in transgenic models harbouring ALS-associated gene mutations have failed to translate to the clinic, suggesting that genetic models may not necessarily reflect pathophysiological mechanisms underlying sporadic amyotrophic lateral sclerosis (sALS).^5,6^ A diverse array of mechanisms including oxidative stress, glutamate excitotoxicity, mitochondrial dysfunction, altered axonal transport, and RNA and protein processing have all been explored as contributors to disease pathogenesis in ALS, but whether these mechanisms have the same degree of importance in different ALS subtypes is unclear.^7^

Prior studies have reported neurotoxic properties of ALS CSF and established the feasibility of using ALS CSF to transfer disease pathology to animals.^8–14^*In vitro* studies have demonstrated induction of neuronal death within 24 h of incubation in ALS CSF,^8^ whereas various *in vivo* studies have reported pathological changes induced by intrathecal ALS CSF injections in neonatal rats,^12^ disruption of motor cortex neuronal activity, and motor impairments following intracerebroventricular infusions in adult rats^13^ and TDP-43 transgenic mice.^14^ However, these previous animal studies did not investigate potential differences in neurotoxicity of CSF from patients with sporadic or familial subtypes of ALS and the identities of neurotoxic candidates in CSF responsible for triggering ALS pathology remain unknown.

In this study, we directly compared the neurotoxic capacity of CSF obtained from sALS patients and familial amyotrophic lateral sclerosis (fALS) patients with mutations in *SOD1*, *C9orf72* and *TARDBP* using a novel CSF-mediated mouse model which can reliably detect motor impairments and pathological changes in the spinal cord induced by a single 3 µL injection of CSF into the cervical subarachnoid space. By performing CSF filtration studies to systematically eliminate components by molecular weight and unbiased global proteome profiling of sALS CSF pre- and post-filtration, we identified apolipoprotein B-100 (ApoB) as the neurotoxic agent responsible for inducing motor disability, motor neuron death, and other hallmark ALS pathology in the spinal cord. Importantly, we show that CSF filtration and targeted depletion of ApoB from sALS CSF prevents the induction of motor deficits and motor neuron degeneration, thus providing a novel therapeutic target and therapeutic approach to alleviate disease pathophysiology in the predominant sporadic form of ALS.

## Materials and methods

### Patient demographics and CSF collection

CSF was obtained from a total of 18 ALS patients, 11 of whom had sALS and seven had fALS (Table 1). All of the ALS patients were diagnosed by board-certified neurologists with a subspeciality interest in ALS. Five of the 11 sALS patients and all seven fALS patients were seen at the Sean M. Healey and AMG Center for ALS at Massachusetts General Hospital in Boston. The other six sALS patients were seen at the Larry G. Gluck Division of ALS Research at the Tisch MS Research Center of New York (Tisch Center). All control samples, except one, were obtained from the CSF bank at the Tisch Center.

**Table 1:** Patient demographics for CSF samples used for cervical subarachnoid injections

	ALS				Controls	
Diagnosis	Sporadic ALS	*SOD1*	*C9*	*TARDBP*	DC	HC
No. of patients (CSF samples)	11	3	3	1	5	4
Gender distribution	7 Males; 4 Females	3 Males	2 Males; 1 Female	1 Female	1 Male; 4 Females	1 Male; 3 Females
Age at sample collection, mean (SD), years	59.2 (8.2)	57.8 (9.2)	58.8 (4.1)	72.5	46.6 (14.1)	53.3 (13.1)
Disease duration at sample collection, mean (SD), years	3.5 (3.9)	1.1	1.4 (1.0)	7.7	6.9 (6.7)	N/A
ALSFRS total score, mean (SD)	37.4 (8.6)	30	36 (6.0)	37	N/A	N/A

DC, disease control; HC, healthy control; ALSFRS, ALS functional rating scale.

CSF samples were collected from patients with ALS, five multiple sclerosis patients as disease controls (DCs), and four healthy control (HC) volunteers following Institutional Review Board approval and informed consent according to the Declaration of Helsinki. None of the multiple sclerosis patients received any kind of immunomodulatory treatment for at least 6 months prior to CSF collection. Samples were collected with sterile techniques either by lumbar puncture or access port aspiration of surgically implanted pumps. CSF samples were centrifuged at 200 x g for 15 min to remove cells, confirmed to be free of red blood cell contamination by microscopy, then stored in aliquots at –80°C.

### Intrathecal injection into cervical subarachnoid space in mice

Adult female C57BL/6J mice (aged 8–12 weeks) purchased from The Jackson Laboratory (Bar Harbor, ME) were used in all *in vivo* experiments. All procedures were approved by the Institutional Animal Care and Use Committee at Mispro Biotech Services (New York). Prior to surgery, mice were anaesthetized with a ketamine (110 mg/kg) and xylazine (10 mg/kg) cocktail and received subcutaneous injections of 0.1 mg/kg buprenorphine, 2.5 mg/kg baytril and 1 ml 0.9% saline. Laminectomies at cervical levels 4 (C4) and 5 (C5) were performed to expose the underlying spinal cord. A 32-gauge Hamilton syringe was inserted underneath the dura mater and 3 µL saline, total CSF, or individual human CSF proteins were slowly injected into the subarachnoid space. Proteins injected included the following: human ApoB purified from plasma (Millipore), human MOG protein 35–55 (Sigma-Aldrich), native human haptoglobin (abcam), human ApoC-III purified from plasma (Athens Research & Technology), recombinant human ApoE (Novus Biologicals) and recombinant human CHIT1 (ABclonal). A minimum of three mice were injected per unpooled, individual patient CSF sample or protein sample. Mice were assigned to different treatment groups in a randomized manner.

### Behavioural testing

#### Motor deficit score testing

Following intrathecal delivery of CSF or proteins, all mice underwent motor testing at 1 day post-injection (DPI). A separate cohort of mice used for time course analysis was tested during six additional sessions until 28 DPI. Forelimb reaching, gripping and tail flaccidity were evaluated on a 3-point scale. Mice were held by their tails above their cage bars and allowed to reach out and grip the bars for five trials. Mice displaying no motor deficits were given a score of 0. Any deficits in either reaching or gripping were each given a score of 1. Specifically, an inaccurate reach was considered to be a reaching deficit, and weakness in grip strength or clenched forepaws were scored as gripping deficits. Tail flaccidity was also given a score of 1. All motor testing was performed blinded with respect to treatment groups.

#### Grip strength testing

Mice were habituated to the grip strength metre (TSE systems) for 3 days prior to surgery. Each mouse was given 1 min to explore the grip strength metre, then held by their tails and allowed to grip the bar with both forelimbs for five consecutive trials. After a 30 s rest period, the mouse was given another five trials to grip and then returned to their home cage. Baseline grip strength force was measured at 1 day prior to surgery and grip strength was also measured at 1 DPI. A separate cohort of mice used for time course analysis was tested during six additional sessions until 28 DPI. The mean grip strength force was calculated from five trials. Normalized grip strength values were calculated by dividing mean grip strength force on post-injection testing days by mean baseline grip strength force.

### Tissue harvesting

Mice were sacrificed using an overdose of ketamine (300 mg/kg) and xylazine (30 mg/kg) and were perfused transcardially with phosphate buffered saline (PBS) followed by 4% paraformaldehyde in 0.1 M PBS, pH 7.4. Spinal cords and brains were dissected out, postfixed in 4% paraformaldehyde overnight and then placed in 30% sucrose overnight for cryoprotection.

Cervical spinal cords were cut 0.5 cm rostral and 0.5 cm caudal to the injection site, and 1 cm of thoracic spinal cords were also collected. The 1 cm segments were then embedded and frozen in Tissue Tek® (VWR International, PA). Spinal cords were sectioned sagittally at 20 µm thickness using a cryostat (Leica) and then slide-mounted onto Histobond® slides (VWR International, PA). The anatomical orientation of tissue sections, as well as the order and position in which they were mounted onto the slides were kept consistent to facilitate unbiased histological comparisons, as described in further detail below. Brains were sectioned coronally at 30 µm and free-floating sections were stored in 0.01% sodium azide in PBS.

### Immunofluorescence staining

Immunostaining was performed on series of spinal cord sections at 100 µm intervals throughout the entire cervical spinal cord or thoracic spinal cord. Slides with spinal cord sections or cells, or free-floating brain sections were washed three times in 0.1% triton X-100 in PBS (PBS/T), then incubated in 10% normal goat serum (NGS) or normal donkey serum (NDS) in PBS/T for 1 h at room temperature. Primary antibodies were diluted in 10% NGS or NDS in PBS/T and incubation occurred overnight at 4°C. After incubation, slides or sections were rinsed three times in PBS and incubated in a 1:750 dilution of the appropriate Alexa-Fluor secondary antibodies (Invitrogen) in 10% NGS or NDS in PBS/T for 1.5 h at room temperature. Slides or sections were rinsed three times in PBS and then counterstained with 1:2500 DAPI in PBS (Invitrogen) for 5 min. After two final washes in PBS, free-floating brain sections were mounted onto slides, and slides were mounted using Fluoromount (Sigma).

Primary antibodies used include the following: goat anti-ChAT (Millipore, 1:100), rabbit anti-activated caspase-3 (R&D, 1:400), mouse anti-GFAP (Novus Biologicals, 1:500), rabbit anti-Iba1 (Wako, 1:500), mouse anti-SMI-32 (Covance, 1:1000), rabbit anti-GLT-1 (Abcam, 1:200), rabbit anti-TDP-43 (Proteintech, 1:250), mouse anti-NeuN (Millipore, 1:200), rabbit anti-Olig2 (Millipore, 1:500), and rabbit anti-Ki67 (Abcam, 1:200).

### Luxol fast blue staining

Slides were washed in PBS and then water before immersing in 70% ethanol, 80% ethanol, then 90% ethanol twice, for 5 min each. Slides were incubated in 0.1% luxol fast blue in 95% alcohol with acetic acid (Electron Microscopy Sciences) at 60°C for 2.5 h. Excess staining was removed by rinsing slides in water. Staining was then differentiated by immersing slides in 0.05% lithium carbonate (ACROS H_2_O Organics). Slides were then stained in 0.1% cresyl violet (Sigma). After rinsing in water, slides were dehydrated in three washes each of 70%, 90% and 100% ethanol for 5 min. Slides were cleared in three washes of xylene for 5 min each and then mounted using cytoseal (Thermo Scientific).

### Histological analyses

Images were captured at 20X magnification using a Zeiss Axio Imager. Acquisition parameters and exposure times were kept consistent for each antibody stain. To ensure unbiased comparisons between experimental groups, spinal cord images were captured from similar tissue section numbers on the slides and matching anatomical regions were verified by experimenters. The number of motor neurons, number of cytoplasmic TDP-43 expressing motor neurons and immunostaining intensities were quantified using ImageJ software. For cell counts, three images were quantified per mouse to calculate the mean motor neuron number or mean number of cytoplasmic TDP-43 expressing motor neurons. Fluorescence intensities were measured as mean grey values in regions of interest. Both imaging and quantification were performed by experimenters blinded for treatment groups.

### CSF filtration studies

sALS CSF samples underwent tangential flow filtration using 5 kDa, 100 kDa, 300 kDa, or 750 kDa MWCO hollow-fibre filters (Spectrum, California). Filters were primed with sterile-filtered distilled H_2_O prior to sample filtration. CSF was passed through the filters at a flow rate of 0.7 mL/min using a peristaltic pump for a total of three filtration cycles (Watson Marlow, Wilmington, MA), then stored in aliquots at –80°C until use. CSF protein concentrations were measured using a Pierce BCA protein assay according to manufacturer instructions (Thermo Fisher Scientific). Unfiltered and filtered sALS CSF samples were injected intrathecally into mice, as described previously.

### Coomassie staining

Unfiltered and filtered CSF samples were run on a NuPAGE™ 4–12% bis-tris (5 and 100 kDa filtrates) or NuPAGE™ 3–8% tris-acetate protein gel (300 and 750 kDa filtrates) (Thermo Fisher Scientific). MagicMark™ XP protein standard was run on the 4–12% gel and HiMark™ protein standard was used for the 3–8% gel (Thermo Fisher Scientific). Gels were placed in 25% isopropanol with 10% acetic acid for 30 min and then stained in 0.006% Coomassie brilliant blue in 10% acetic acid overnight at room temperature. Following destaining with 10% acetic acid, gels were rinsed in H_2_O. Images of gels were captured using Carestream MI system and software.

### Proteomic analysis of human CSF

#### Sample preparation

sALS CSF (*n* = 5), 5 kDa-filtered sALS CSF (*n* = 2), multiple sclerosis CSF (*n* = 5) and HC CSF (*n* = 5) samples were shipped frozen to Biognosys for label-free unbiased global proteome profiling using hyper reaction monitoring mass spectrometry (HRM™/DIA-MS)(Biognosys AG, Switzerland). In total, 100 µL per CSF sample was denatured using Biognosys’ Denature Buffer, reduced/alkylated using Biognosys’ Reduction/Alkylation Solution for 60 min at 37°C in the dark and then digested into peptides using 1 µg of trypsin (Promega) per sample overnight at 37°C.

#### Clean-up for mass spectrometry

Peptides were desalted using BioPureSPN Midi columns (The Nest Group) according to the manufacturer’s instructions and dried down using a SpeedVac system. Peptides were resuspended in 20 µL LC solvent A [1% acetonitrile, 0.1% formic acid (FA)] and spiked with Biognosys’ iRT kit calibration peptides. Peptide concentrations were determined using a UV/VIS Spectrometer (SPECTROstar Nano, BMG Labtech).

#### H**igh-pH reversed-phase** fractionation

Peptides from all analyzed CSF samples were used to generate a sample pool. Ammonium hydroxide was added to a pH value > 10. The fractionation was performed using a Dionex UltiMate 3000 RS pump (Thermo Scientific) on an Acquity UPLC CSH C18 1.7 µm, 2.1 × 150 mm column (Waters). The gradient was 1–40% solvent B in 20 min, solvents were A: 20 mM ammonium formate in water, B: acetonitrile. Fractions were taken every 30 s and sequentially pooled to eight fractions. These were dried down and resolved in 12 µL solvent A. Prior to mass spectrometric analyses, they were spiked with Biognosys’ iRT kit calibration peptides. Peptide concentrations were determined using a UV/VIS Spectrometer (SPECTROstar Nano, BMG Labtech).

#### Shotgun mass spectrometry acquisition

For DDA LC-MS/MS measurements, 1 µg of peptides per sample were injected to an in-house packed reversed phase column (PicoFrit emitter with 75 µm inner diameter, 60 cm length and 10 µm tip from New Objective, packed with 1.7 µm Charged Surface Hybrid C18 particles from Waters) on a Thermo Scientific™ EASY-nLC™ 1200 nano-liquid chromatography system connected to a Thermo Scientific™ Q Exactive™ HF mass spectrometer equipped with a Nanospray Flex™ Ion Source. A modified TOP15 method was used. The full-range MS1 scan was followed by 15 data-dependent MS2 scans.

#### HRM™ mass spectrometry acquisition

HRM™ MS was performed for each individual CSF sample. For DIA LC-MS/MS measurements, 1 µg of peptides per sample were injected to an in-house packed reversed phase column, as described previously. The DIA method consisted of one full-range MS1 scan and 29 DIA segments.

#### Database search of LC-MS/MS data

Shotgun and HRM™ mass spectrometric data were analyzed using SpectroMine™ software (Biognosys). The false discovery rate on peptide and protein level was set to 1%. Data were searched against a human UniProt protein database (Homo sapiens, 2020-01-01). Search results were used to generate a shotgun-based spectral library of HPRP fractions and an HRM™-based spectral library of all samples. SpectroMine™ was then used to combine the separate libraries into a hybrid, which was subsequently used for HRM™ data quantification.

#### HRM^™^ mass spectrometry data analysis

HRM™ mass spectrometric data were analyzed with Spectronaut® Pulsar software (Biognosys). Differentially regulated proteins were identified using the following criteria: q-value < 0.05 and average fold change > 1.5. The false discovery rate on peptide and protein level was set to 1%, and data were filtered using row-based extraction. Data were normalized using local regression normalization. For testing of differential protein abundance, protein intensities were analyzed using a two-sample Student’s *t*-test. Gene Ontology (GO) enrichment analysis was performed in Spectronaut® software using a human gene associations file obtained from EBI (2018-02-20). Only terms with a minimum of two members were considered.

### ApoB and IgG depletion

ApoB antibody (Invitrogen) was incubated with Dynabeads® Protein A (Thermo Fisher Scientific) for 45 min at room temperature. Antibody-coated Dynabeads® were then incubated with sALS CSF for 2 h at 4°C. IgG depletion was performed with uncoated Dynabeads®. ApoB or IgG-depleted sALS CSF were centrifuged in an ultrafree-MC centrifugal filter tube at 12 000 x g at 4°C for 6 min. Samples were stored at –80°C until use.

### ApoB ELISA

ApoB protein in sALS, SOD1, C9, TARDBP, HC, multiple sclerosis, and ApoB-depleted sALS CSF was quantified using a human ApoB ELISA kit (Thermo Fisher Scientific) according to the manufacturer’s instructions.

### Human astrocyte cultures

Human cortical astrocytes were purchased from Lonza and plated at a density of 10 000 cells/cm^2^ in either 6-well plates for RNA extraction, or 8-well chamber slides for immunocytochemistry. Astrocytes were cultured in AGM™ astrocyte growth medium (Lonza) containing 3% fetal bovine serum until they reached 80–90% confluence. Astrocytes were treated with either CSF diluted at a ratio of 1:1 in serum-free DMEM, or 0.01 µg/µL or 0.01 ng/µL human ApoB protein (Millipore), human MOG protein (Sigma-Aldrich), or human haptoglobin protein (abcam) in DMEM. After 24 h, astrocytes were either processed for RNA extraction using a RNeasy Plus Micro Kit (Qiagen, Venlo, Netherlands) according to manufacturer instructions, or fixed with 4% paraformaldehyde for immunocytochemistry. Using ImageJ, Ki67^+^ and GFAP^+^ astrocyte numbers were quantified on a minimum of three images per well and then averaged.

### Human iPSC-derived motor neurons

Human induced pluripotent stem cell (iPSC)-derived motor neurons were purchased from iXCells Biotechnologies and plated at a density of ∼86 000 cells/cm^2^ in 8-well chamber slides coated with 80 µg/µL Matrigel in DMEM/F-12. Motor neurons were cultured in Motor Neuron Maintenance Medium (iXCells Biotechnologies) for 8 days. On Day 8, human ApoB (Millipore), human MOG (Sigma-Aldrich), human haptoglobin (abcam), human ApoC-III (Athens Research & Technology), and human ApoE (Novus Biologicals) proteins were applied at 0.01 µg/µL or 0.05 µg/µL in media, or 50% sALS CSF and ApoB-depleted sALS CSF diluted in media were applied. In a separate ApoB titration experiment, lower doses of 0.005 ng/µL or 0.01 ng/µL ApoB were applied. At 24 h pre- and post-treatment, cells were visualized using a 10X objective on an Olympus IX71 microscope and bright field images were captured on a Hamamatsu Orca-Spark camera using Cell Sens Dimension Programme. Motor neurons were fixed with 4% PFA for immunocytochemistry at 24 h post-treatment. Using ImageJ, areas of ChAT^+^ motor neuron clusters were quantified from three images per well and then averaged.

### Quantitative real-time PCR

Quantitative real-time PCR (q-RT-PCR) was performed using the Applied Biosystems QuantStudio 7 Flex Real-Time PCR system with individual TaqMan probes (Applied Biosystems, Foster City, CA). The ΔΔCt method was used to calculate fold changes in RNA expression, with the DMEM group as the calibrator and β-actin as the internal control.

### Statistical analyses

GraphPad Prism 8 software was used to perform statistical analyses. Motor deficit scores, normalized grip strength, number of motor neurons and astrocytes, percentage of cytoplasmic TDP-43^+^ motor neurons, size of motor neuron clusters, and immunostaining intensities were analyzed using a one-way ANOVA, with Bonferroni *post hoc* analyses. Motor deficit scores and normalized grip strength for the 28 DPI experiment were analyzed using a repeated measures two-way ANOVA, with Bonferroni *post hoc* analyses. Protein concentrations and ELISA measurements of CSF ApoB levels were analyzed using a paired, two-tailed Student’s *t*-test. Statistical significance was determined by *P* < 0.05. Data are presented as mean ± SEM.

### Data availability

Data reported in this manuscript are available from the corresponding author, upon reasonable request.

## Results

### sALS CSF but not fALS CSF induces persistent motor disability and motor neuron degeneration in mice

**Figure 1:**
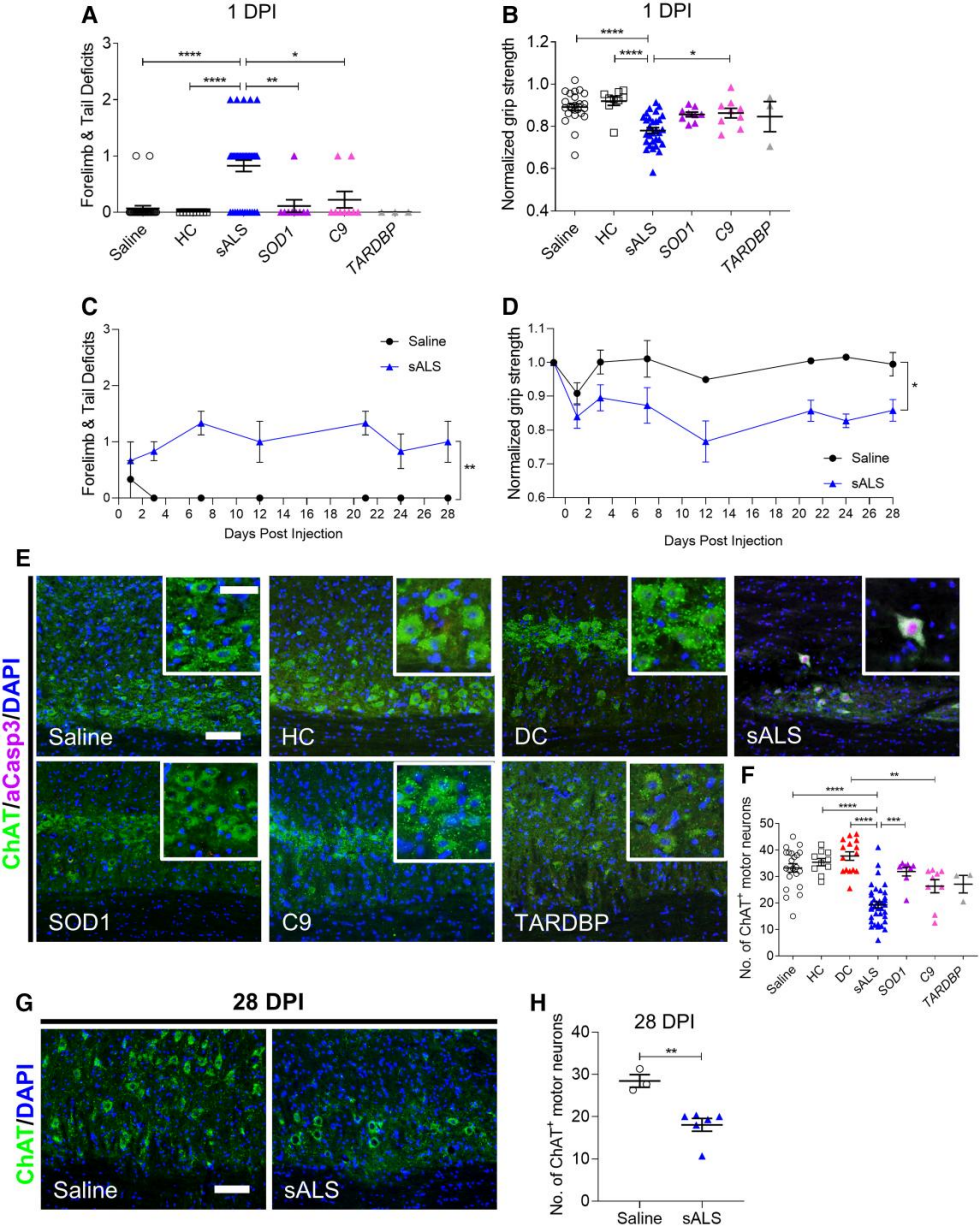
**Motor deficits and motor neuron degeneration are induced by CSF from sALS but not fALS patients.** (**A**) Motor deficit scores and (**B**) normalized forelimb grip strength at 1 day post-intrathecal delivery of saline, CSF from HC (*n* = 3), patients with sALS (*n* = 11), *SOD1* ALS (*n* = 3), *C9orf72* (C9) ALS (*n* = 3), or *TARDBP* ALS (*n* = 1). Each CSF sample was injected into a minimum of 3 mice. Saline (*n* = 29 mice), HC (*n* = 9 mice), sALS (*n* = 40 mice), SOD1 (*n* = 9 mice), C9 (*n* = 9 mice), TARDBP (*n* = 3 mice). (**C**) 28-day time course of motor deficit scores and (**D**) normalized forelimb grip strength following intrathecal injections of saline or sALS CSF (*n* = 2). Saline (*n* = 3 mice), sALS (*n* = 6 mice). (**E**) Representative images of cervical spinal cords immunostained for ChAT and activated caspase-3 at 1 DPI of saline, HC CSF (*n* = 4), CSF from multiple sclerosis patients as DCs (*n* = 5), patients with sALS (*n* = 11), *SOD1* ALS (*n* = 3), *C9* ALS (*n* = 3), or *TARDBP* ALS (*n* = 1). Scale bar, 100 µm, inset: 50 µm. (**F**) Quantification of the number of ChAT^+^ motor neurons in cervical ventral horns at 1 DPI. Saline (*n* = 22 mice), HC (*n* = 10 mice), DC (*n* = 15 mice), sALS (*n* = 37 mice), SOD1 (*n* = 8 mice), C9 (*n* = 9 mice), TARDBP (*n* = 3 mice). (**G**) Representative images of ChAT immunostaining in cervical spinal cords at 28 DPI of saline or sALS CSF (*n* = 2). Scale bar, 100 µm. (**H**) Quantification of the number of ChAT^+^ motor neurons in cervical ventral horns at 28 DPI. Saline (*n* = 3 mice), sALS (*n* = 6 mice). Data plotted as mean ± SEM. Each point represents an individual mouse (**A, B, F** and **H**). One-way ANOVA (**A, B,** and **F**) or repeated measures two-way ANOVA (**C** and **D**) with Bonferroni’s test. Unpaired *t*-test (**H**). ****P < 0.0001, ***P < 0.001, **P < 0.01, *P < 0.05.

Forelimb motor function was assessed following intrathecal CSF injection into the mid-cervical subarachnoid space of adult female C57BL/6J wild-type mice. Mice injected with sALS CSF developed weakness of forelimb grip strength, impaired forelimb reach and tail flaccidity by 1 DPI. Surprisingly, mice injected with CSF obtained from patients with *SOD1*, *C9orf72* or *TARDBP* mutations did not exhibit motor deficits and performed similarly to control mice injected with saline or CSF from HC (Fig. 1A and B). We then tested whether motor disability induced by the 3 µL sALS CSF injection was transient since CSF turnover in the mouse occurs approximately every 2 h.^15^ A separate cohort of sALS CSF-injected mice tested for 28 days after intrathecal delivery consistently exhibited a greater extent of motor disability relative to saline controls over the entire testing period (Fig. 1C and D).

To assess whether the motor disability in sALS CSF-injected mice was associated with motor neuron degeneration, histological analyses of the cervical spinal cord were performed at 1 and 28 DPI. A significant loss of ChAT^+^ motor neurons was observed in the ventral horns of the cervical spinal cord in sALS CSF-injected mice, as compared with mice injected with saline, HC CSF or SOD1 CSF at 1 DPI (Fig. 1E and F). Surviving motor neurons in sALS CSF-injected mice expressed activated caspase-3, indicating that apoptosis was underway in these cells (Fig. 1E). As a neurological DC, 1 DPI spinal cord tissue from a prior study in which mice had been injected with CSF from multiple sclerosis patients was analyzed. No evidence of motor neuron loss was observed in DC CSF-injected mice. Although motor neuron numbers were reduced in C9orf72 and TARDBP CSF-injected mice, the loss was not as extensive as in sALS CSF-injected mice, and differences only reached statistical significance between the C9orf72 and DC groups. In the 28 DPI cohort, significant motor neuron loss persisted in sALS CSF-injected mice relative to saline controls (Fig. 1G and H). The pathogenic effects of sALS CSF were not restricted to the injection site, as motor neuron loss at 1 DPI extended to the thoracic spinal cord and upper motor neurons in the motor cortex of sALS CSF-injected mice. These results underscore the toxicity of sALS CSF and confirms that our cervical subarachnoid space injection paradigm enables circulation of injected CSF throughout the CNS (Supplementary Fig. 1).

### Pathological TDP-43 cytoplasmic translocation in sALS CSF-injected mice

**Figure 2:**
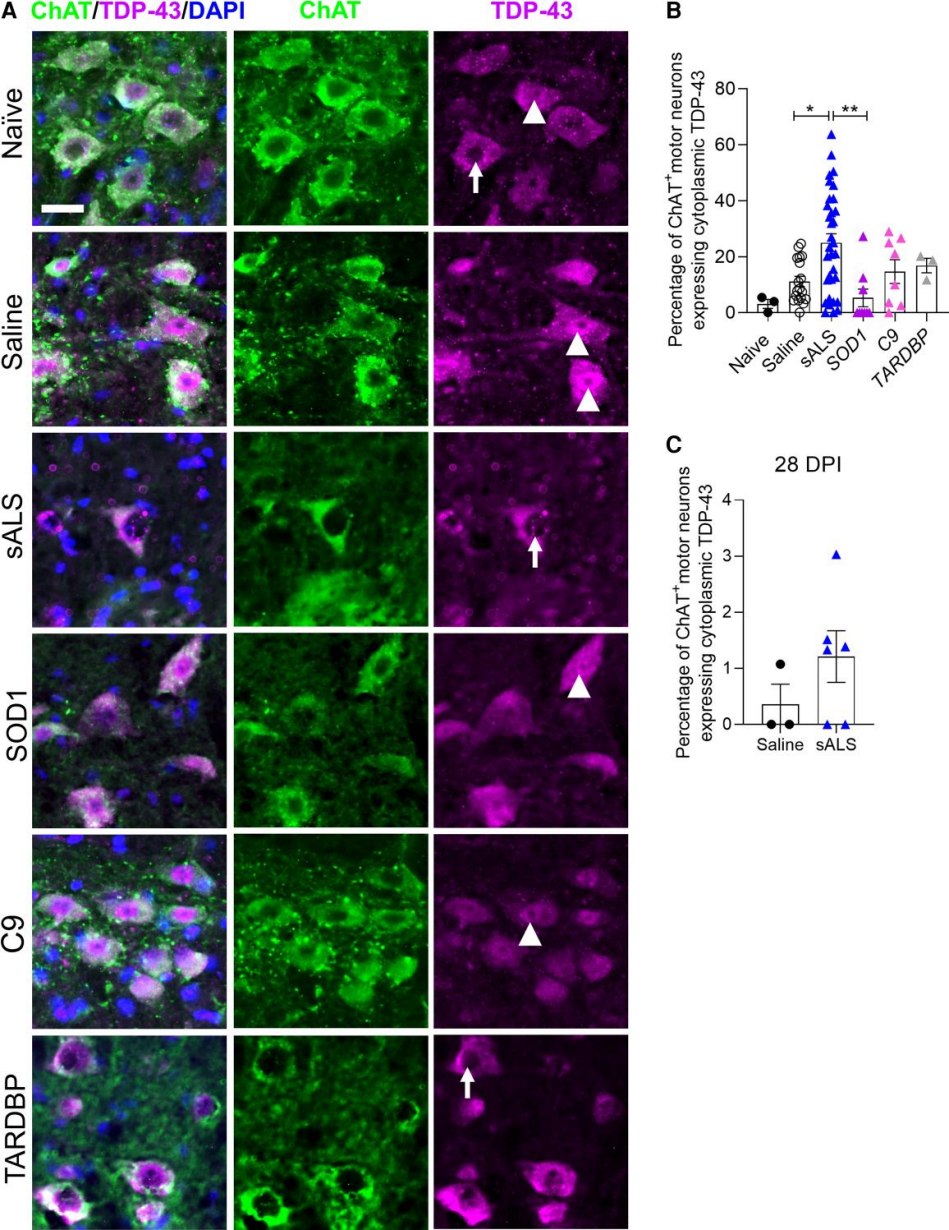
**Pathological translocation of TDP-43 to the cytoplasm is induced by sALS CSF.** (**A**) Representative images of TDP-43 immunostaining in ChAT^+^ motor neurons in naïve mice, and at 1 day following intrathecal delivery of saline, or CSF from sALS (*n* = 11), *SOD1* (*n* = 3), *C9* (*n* = 3), or *TARDBP* (*n* = 1) ALS patients. White arrowheads indicate nuclear TDP-43 expression and white arrows indicate cytoplasmic TDP-43 expression. Scale bar, 25 µm. (**B** and **C**) Quantification of the number of ChAT^+^ motor neurons displaying TDP-43 exclusively in the cytoplasm at 1 DPI (**B**) and 28 DPI (**C**). 1 DPI: Naïve (*n* = 3 mice), saline (*n* = 18 mice), sALS (*n* = 33 mice), SOD1 (*n* = 9 mice), C9 (*n* = 8 mice), TARDBP (*n* = 3 mice). 28 DPI: Saline (*n* = 3 mice), sALS (*n* = 6 mice). Data plotted as mean ± SEM. One-way ANOVA with Bonferroni’s test (**B**). Unpaired *t*-test (**C**). **P < 0.01, *P < 0.05.

The persistent clinical symptoms and motor neuron degeneration observed in sALS CSF-injected mice prompted us to determine if other classically associated hallmarks of ALS pathology^16–24^ were also present. TDP-43 is a DNA/RNA binding protein which translocates to the cytoplasm and disrupts RNA metabolism in the majority of ALS patients.^16,17^ To test whether this also occurred in our disease model, the number of ChAT^+^ motor neurons expressing TDP-43 exclusively in the cytoplasm was quantified in the cervical spinal cord. In naïve uninjected mice, a low baseline percentage of motor neurons express TDP-43 in the cytoplasm. Pathological translocation of TDP-43 occurred in a significant number of motor neurons in sALS CSF-injected mice at 1 DPI, while mice injected with C9orf72 or TARDBP CSF showed only a modest increase in TDP-43 translocation and SOD1 CSF-injected mice showed less translocation compared with saline-injected mice (Fig. 2A and B). The modest increase in TDP-43 translocation in C9orf72 and TARDBP CSF-injected mice suggests that pathological TDP-43 inclusions in *C9orf72* and *TARDBP* patients are induced by non-CSF-mediated mechanisms, and the lack of TDP-43 translocation in SOD1 CSF-injected mice is consistent with the absence of TDP-43^+^ inclusions in ALS patients with *SOD1* mutations.^18^ At 28 DPI, cytoplasmic TDP-43 expression was higher but not significantly in sALS CSF-injected mice compared with saline controls, and overall cytoplasmic TDP-43 levels in both groups were lower than at 1 DPI (Fig. 2C).

### sALS CSF triggers neurofilament-H and glutamate transporter-1 upregulation

**Figure 3:**
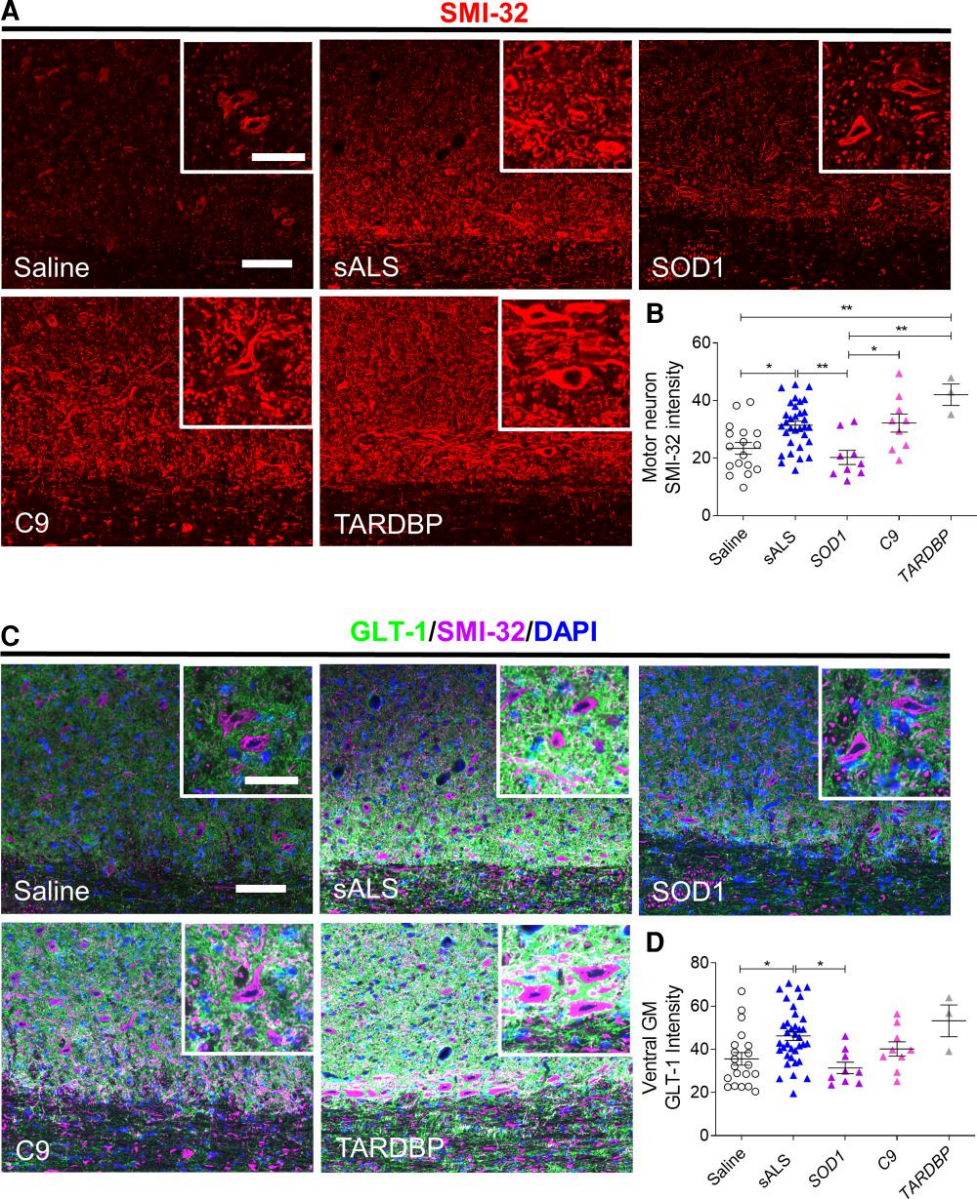
**NF-H and glutamate transporter-1 upregulation occurs in cervical ventral horns at 1 day post-sALS CSF administration.** (**A** and **C**) Representative images of cervical spinal cords immunostained for nonphosphorylated NF-H (SMI-32) and glutamate transporter-1 (GLT-1) at 1 DPI of saline, sALS CSF (*n* = 11), SOD1 CSF (*n* = 3), C9 CSF (*n* = 3), or TARDBP CSF (*n* = 1). Scale bar, 100 µm, inset: 50 µm. (**B** and **D**) Quantification of SMI-32 (**B**) or GLT-1 (**D**) immunostaining intensities at 1 DPI in areas surrounding motor neurons or ventral grey matter, respectively. Saline (*n* = 17 mice), sALS (*n* = 32 mice), SOD1 (*n* = 9 mice), C9 (*n* = 9 mice), TARDBP (*n* = 3 mice). Data plotted as mean ± SEM. Each point represents an individual mouse (**B** and **D**). One-way ANOVA with Bonferroni’s test. **P < 0.01, *P < 0.05.

Studies of post-mortem spinal cord tissue from ALS patients have reported elevated expression of neurofilament-H (NF-H) in degenerating motor neurons.^19^ We assessed whether intrathecal delivery of ALS CSF in mice could also induce similar pathology in regions surrounding degenerating motor neurons. Levels of NF-H expression, as measured by SMI-32 immunostaining intensity, were significantly upregulated in mice injected with sALS, C9orf72 or TARDBP CSF compared with saline and SOD1 CSF-injected mice (Fig. 3A and B). These findings are consistent with the lower numbers of motor neurons observed and suggests the occurrence of neuronal damage in the cervical spinal cord.

Since glutamate excitotoxicity has been implicated as one of the pathological mechanisms underlying motor neuron degeneration in ALS,^20,21^ we explored whether ALS CSF-induced NF-H upregulation was associated with changes in glutamate transporter-1 (GLT-1) expression. GLT-1 immunostaining in the cervical spinal cord revealed significant upregulation of GLT-1 in the ventral horns of sALS CSF-injected mice compared to saline and SOD1 CSF-injected mice, perhaps as a compensatory response to sALS CSF-induced excitotoxicity (Fig. 3C and D). GLT-1 expression was also higher in C9 and TARDBP CSF-injected mice, although statistical significance was not reached.

### sALS CSF induces reactive astrogliosis and microglial activation

**Figure 4:**
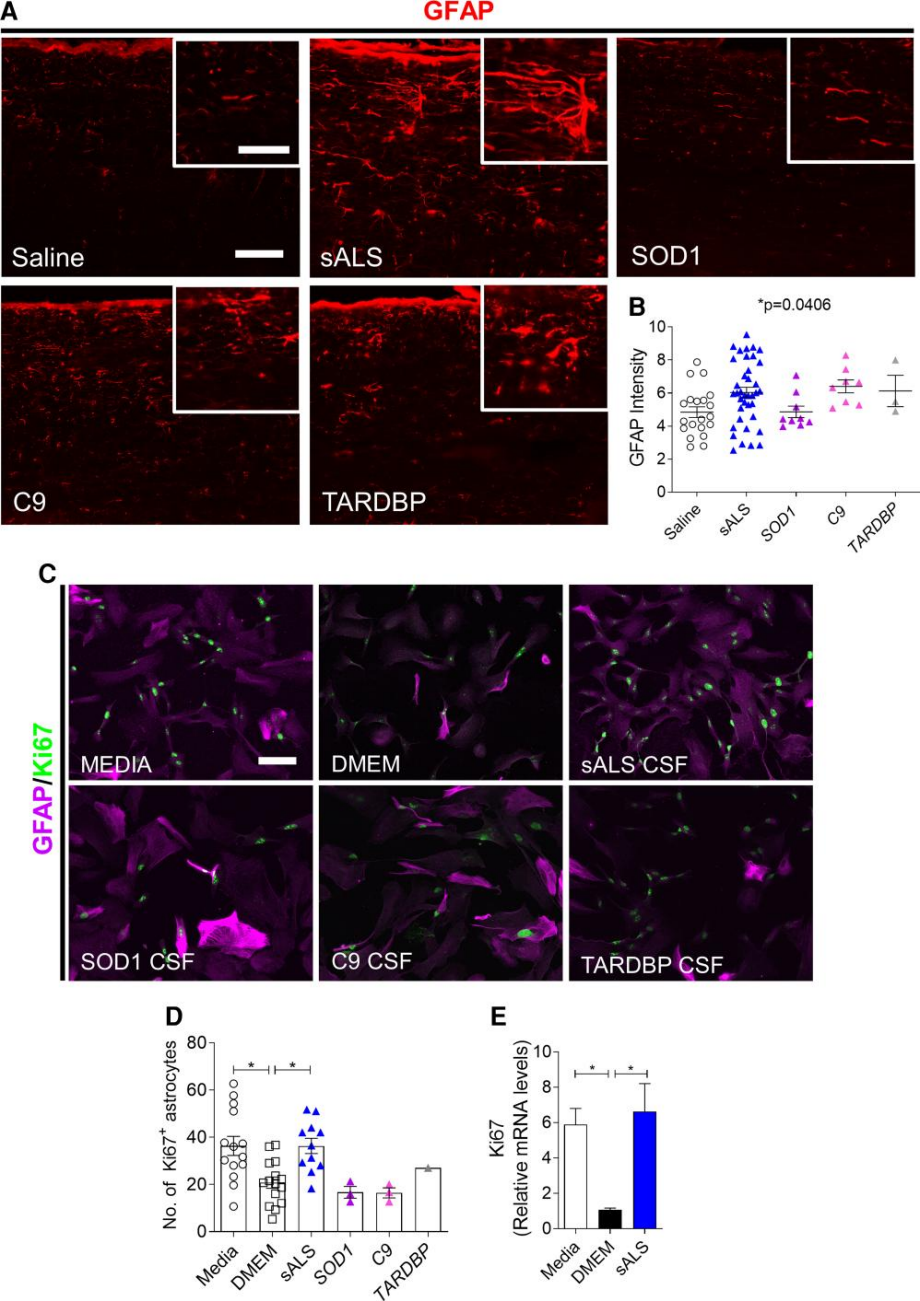
**sALS CSF induces GFAP upregulation in mice and proliferation of primary human astrocyte cultures.** (**A**) Representative images of cervical spinal cords immunostained for GFAP at 1 DPI of saline, sALS CSF (*n* = 11), *SOD1* CSF (*n* = 3), *C9orf72* (C9) CSF (*n* = 3), or *TARDBP* CSF (*n* = 1). Scale bar, 100 µm, inset: 50 µm. (**B**) Quantification of GFAP immunostaining intensity in dorsal white matter at 1 DPI. Saline (*n* = 20 mice), sALS (*n* = 38 mice), SOD1 (*n* = 9 mice), C9 (*n* = 8 mice), TARDBP (*n* = 3 mice). Data plotted as means ± SEM. Each point represents an individual mouse. (**C**) Representative images of human primary astrocytes cultured in media for 5 days and then incubated for 24 h with DMEM or 50% sALS CSF, SOD1 CSF, C9 CSF, or TARDBP CSF diluted in DMEM. Scale bar, 100 µm. (**D**) Quantification of the number of Ki67^+^ proliferating human astrocytes following 24 h 50% CSF treatment. (**E**) Ki67 mRNA levels in human astrocytes following 24 h exposure to 50% CSF, as determined by qPCR. One-way ANOVA with Bonferroni’s test. *P < 0.05.

As GLT-1 is predominantly expressed on astrocytes, we next investigated whether sALS CSF-induced GLT-1 upregulation was also accompanied by reactive astrogliosis, another pathological hallmark observed in ALS.^22^ GFAP immunostaining intensity in the cervical spinal cord revealed higher GFAP expression in mice injected with sALS, C9orf72 and TARDBP CSF, indicating induction of reactive astrogliosis (Fig. 4A and B). To confirm whether ALS CSF could also trigger a similar response in human cells, primary human astrocytes were incubated with 50% CSF in DMEM for 1 day, and proliferation was assessed by quantifying the number of GFAP^+^Ki67^+^ cells and Ki67 mRNA levels. Increased astrocyte proliferation, as indicated by Ki67 protein and mRNA upregulation, was induced only by sALS CSF but not fALS CSF (Fig. 4C-E).

**Figure 5:**
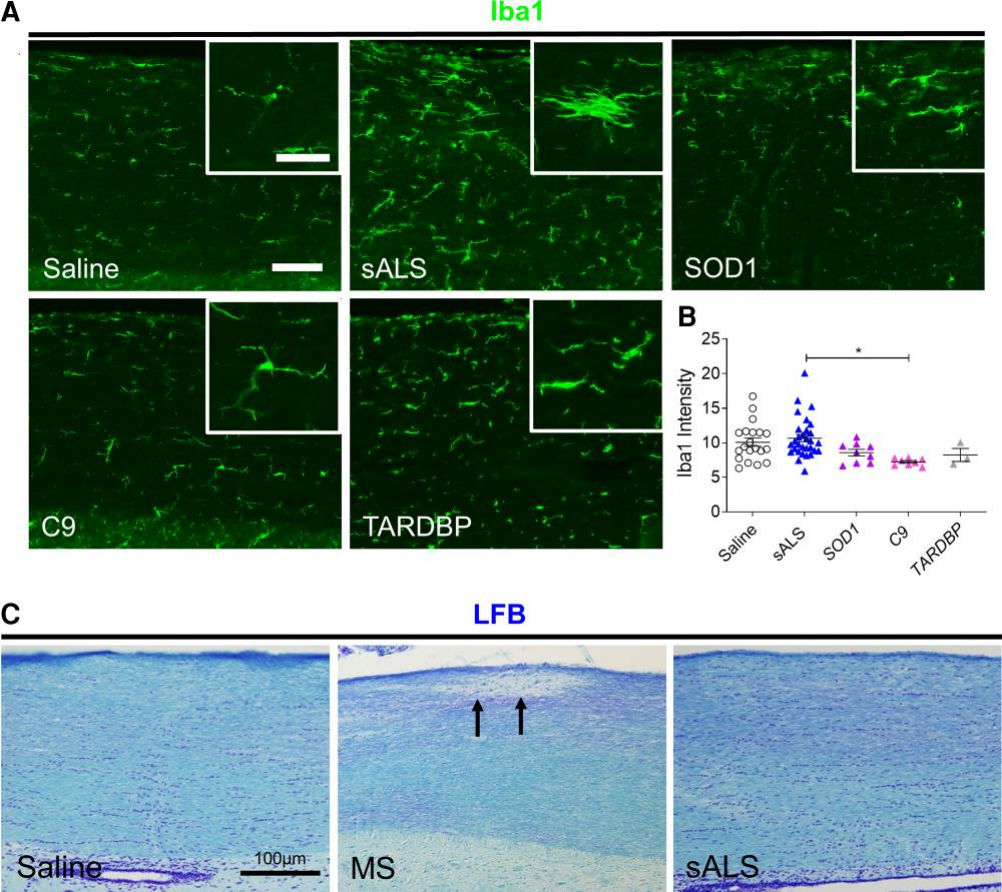
**sALS CSF triggers microglial activation but not demyelination at 1 DPI.** (**A**) Representative images of Iba1 immunostaining in cervical spinal cords at 1 DPI of saline, sALS CSF (*n* = 11), SOD1 CSF (*n* = 3), C9 CSF (*n* = 3), or TARDBP CSF (*n* = 1). Scale bar, 100 µm, inset: 50 µm. (**B**) Quantification of Iba1 immunostaining intensity in dorsal white matter at 1 DPI. Saline (*n* = 20 mice), sALS (*n* = 30 mice), SOD1 (*n* = 9 mice), C9 (*n* = 8 mice), TARDBP (*n* = 3 mice). Data plotted as mean ± SEM. Each point represents an individual mouse. One-way ANOVA with Bonferroni’s test. *P < 0.05. (**C**) Luxol fast blue staining in dorsal white matter of cervical spinal cords. Arrows indicate demyelinated areas. Scale bar, 100 µm.

Microglial activation has also been associated with motor neuron degeneration in ALS.^23,24^ To assess whether microglial activation occurs following intrathecal injection of ALS CSF, cervical spinal cords were immunostained for Iba1. Iba1 immunostaining revealed the presence of activated microglia with characteristic amoeboid morphology in sALS CSF-injected mice, which was absent in all other experimental groups (Fig. 5A). Quantification of Iba1 immunostaining intensity also confirmed sALS CSF-induced microglial activation, with significantly higher Iba1 expression in sALS CSF-injected mice compared with C9 CSF-injected mice (Fig. 5B).

To exclude the possibility that the motor deficits observed in sALS CSF-injected mice were due to demyelination of axons in the cervical spinal cord, luxol fast blue staining was performed to examine myelin integrity. Demyelinated lesions were only observed in multiple sclerosis CSF-injected mice, as would be anticipated, but no areas of demyelination were found in sALS CSF-injected mice, supporting the disease specificity of our findings (Fig. 5C). Together, our results indicate that CSF neurotoxicity differs between various subtypes of ALS but only sALS CSF consistently induces motor weakness in conjunction with the myriad of pathologic findings associated with ALS.

### Filtration and global proteome profiling of sALS CSF implicates ApoB in disease pathogenesis

**Figure 6:**
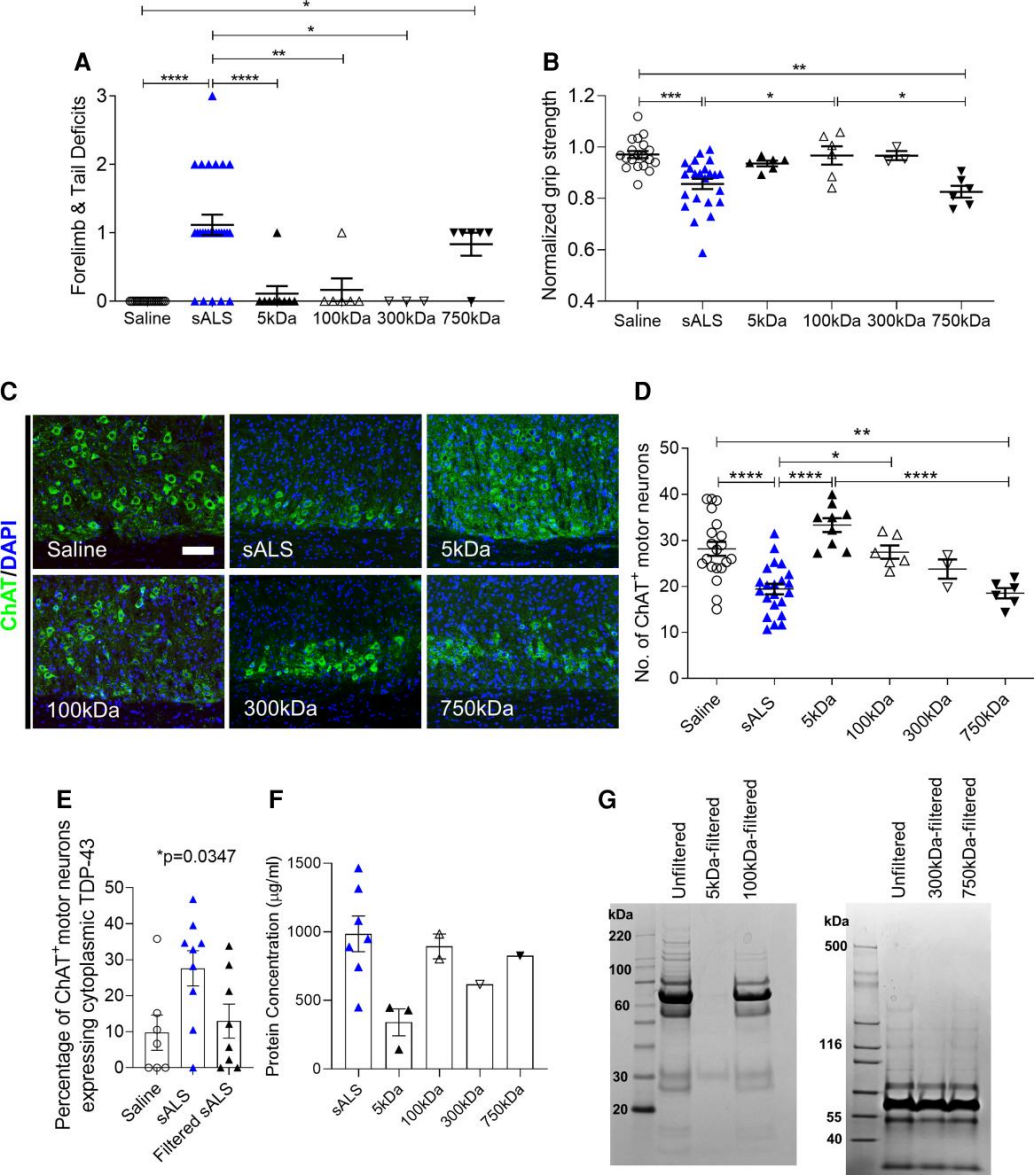
**Neurotoxic components responsible for inducing motor deficits and death of motor neurons are between 300 and 750 kDa.** (**A** and **B**) Motor deficit scores and normalized forelimb grip strength at 1 DPI of saline (*n* = 20 mice), sALS CSF (*n* = 3 samples; *n* = 26 mice), or 5 kDa (*n* = 3 samples; *n* = 9 mice), 100 kDa (*n* = 2 samples; *n* = 6 mice), 300 kDa (*n* = 1 sample; *n* = 3 mice), or 750 kDa-filtered sALS CSF (*n* = 2 samples; *n* = 6 mice). (**C**) Representative images of cervical spinal cords immunostained for ChAT at 1 DPI of saline, sALS CSF, or 5, 100, 300, or 750 kDa-filtered sALS CSF. Scale bar, 100 µm. (**D**) Quantification of the number of ChAT^+^ motor neurons in cervical ventral horns at 1 DPI of unfiltered or filtered sALS CSF. Saline (*n* = 20 mice), sALS (*n* = 22 mice), 5 kDa (*n* = 9 mice), 100 kDa (*n* = 6 mice), 300 kDa (*n* = 3 mice), 750 kDa (*n* = 6 mice). (**E**) Quantification of the number of ChAT^+^ motor neurons displaying TDP-43 exclusively in the cytoplasm at 1 DPI of saline (*n* = 7 mice), sALS CSF (*n* = 9 mice) or 5 kDa-filtered sALS CSF (*n* = 8 mice). (**F** and **G**) CSF protein concentration (**F**) and Coomassie blue staining (**G**) of unfiltered sALS CSF and sALS CSF filtered through 5 kDa, 100 kDa, 300 kDa, or 750 kDa MWCO tangential flow hollow-fibre filters. Data plotted as mean ± SEM. Each point represents an individual mouse (**A, B, D** and **E**) or CSF sample (**F**). One-way ANOVA with Bonferroni’s test. ****P < 0.0001, ***P < 0.001, **P < 0.01, *P < 0.05.

**Figure 7:**
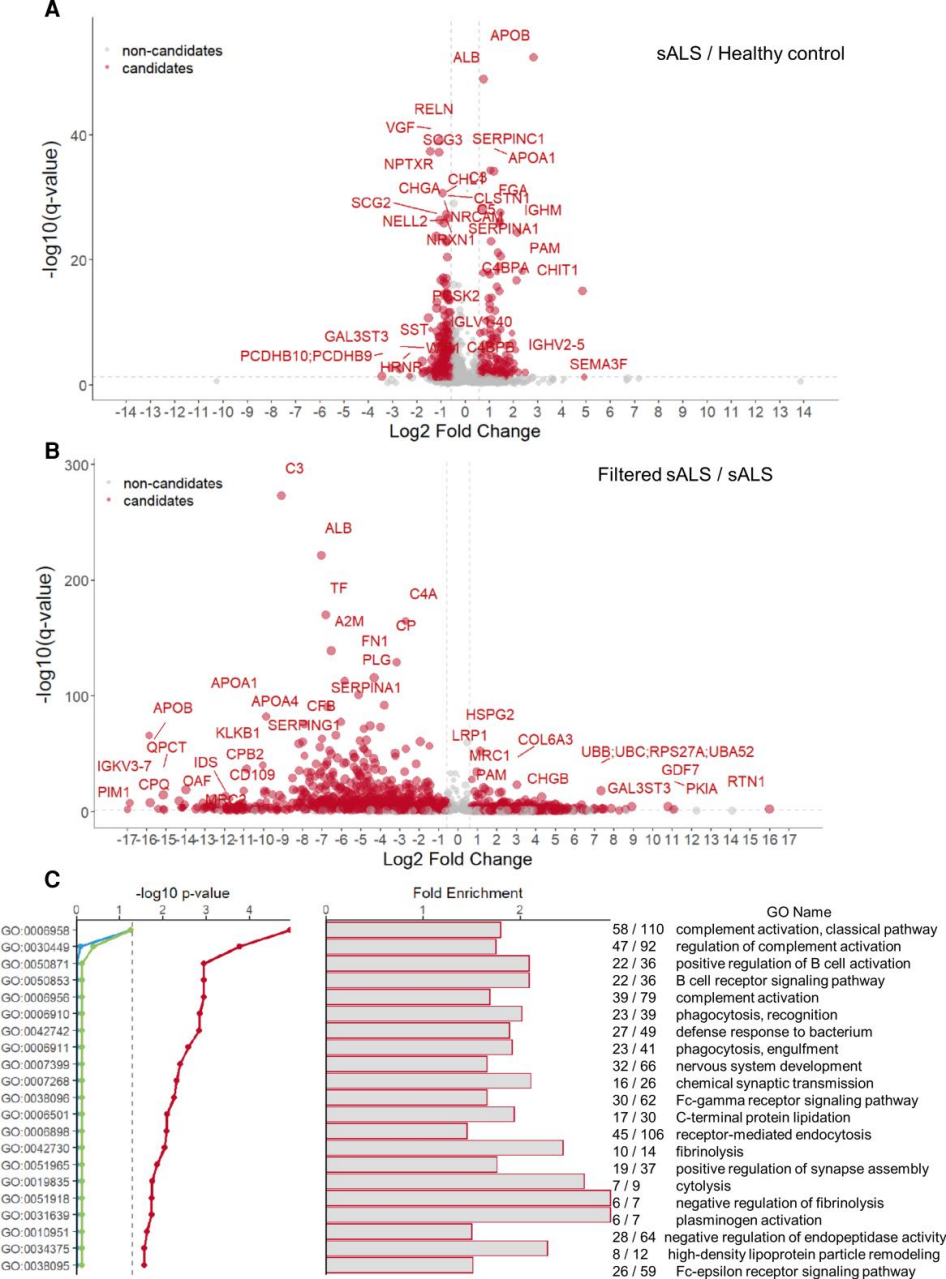
**Global proteome profiling of sALS CSF pre- and post-filtration.** (**A**) Volcano plots showing 1618 proteins quantified in CSF using HRM™ MS, with 490 differentially abundant proteins between sALS CSF (*n* = 5) and HC CSF (*n* = 5). (**B**) 1181 differentially abundant proteins between 5 kDa-filtered sALS CSF (*n* = 2), and sALS CSF (*n* = 5). Red dots represent protein candidates and grey dots represent non-candidates. Differentially regulated proteins were identified using the following criteria: q-value < 0.05 and average fold change > 1.5. (**C**) Top 20 enriched biological process terms from GO enrichment analysis of 490 proteins significantly changed between sALS and HC CSF (*P* < 0.05). Red line represents enrichment *P*-value, green line represents enrichment *P*-value (Benjamini-Hochberg corrected), and blue line represents enrichment *P*-value (Bonferroni corrected).

To identify neurotoxic component(s) circulating in sALS CSF responsible for triggering and/or exacerbating motor neuron degeneration, sALS CSF was filtered to narrow down putative factor(s) by molecular weight. After tangential flow filtration through a 5 kDa MWCO filter, the filtered sALS CSF no longer induced an increase in motor deficit scores or weaker forelimb grip strength, and neither motor neuron loss nor TDP-43 translocation were observed (Fig. 6A-E). Furthermore, mice injected with heat-inactivated sALS CSF did not exhibit motor disability or motor neuron loss, suggesting that heat-sensitive proteins conferred neurotoxicity (Supplementary Fig. 2). We next performed unbiased global proteomic analysis of CSF to search for candidate proteins significantly upregulated in sALS compared with HC and multiple sclerosis CSF, which are also downregulated following filtration. A total of 1618 proteins, represented by 33 010 peptide ion variants, were quantified across all samples analyzed. Four hundred and ninety proteins were found to be differentially abundant between sALS and HC CSF, and 832 of 1609 proteins quantified in sALS CSF were significantly reduced following 5 kDa MWCO filtration (Fig. 7A and B). GO enrichment analysis revealed significant changes in sALS CSF related to biological processes involving complement and immune signalling pathways, synapses and lipids (Fig. 7C).

To further refine the list of putative neurotoxic protein(s) in sALS CSF, we performed additional filtration studies with larger filters until we obtained filtrate which retained neurotoxic protein(s) with the capacity to induce motor deficits and pathology in mice. We found the neurotoxic capacity of both the 100 and 300 kDa filtrates to be attenuated as neither were able to impair motor function or trigger motor neuron degeneration, while the 750 kDa filtrate induced a similar extent of impairment as unfiltered sALS CSF at 1 DPI (Fig. 6A-E). Attenuation of neurotoxicity cannot be attributed to a general reduction in protein concentration, as this was not significantly lowered in the 100 and 300 kDa filtrates (Fig. 6F and G). Taken together, our data suggested that the putative neurotoxic protein(s) are between 300 and 750 kDa in size.

**Figure 8:**
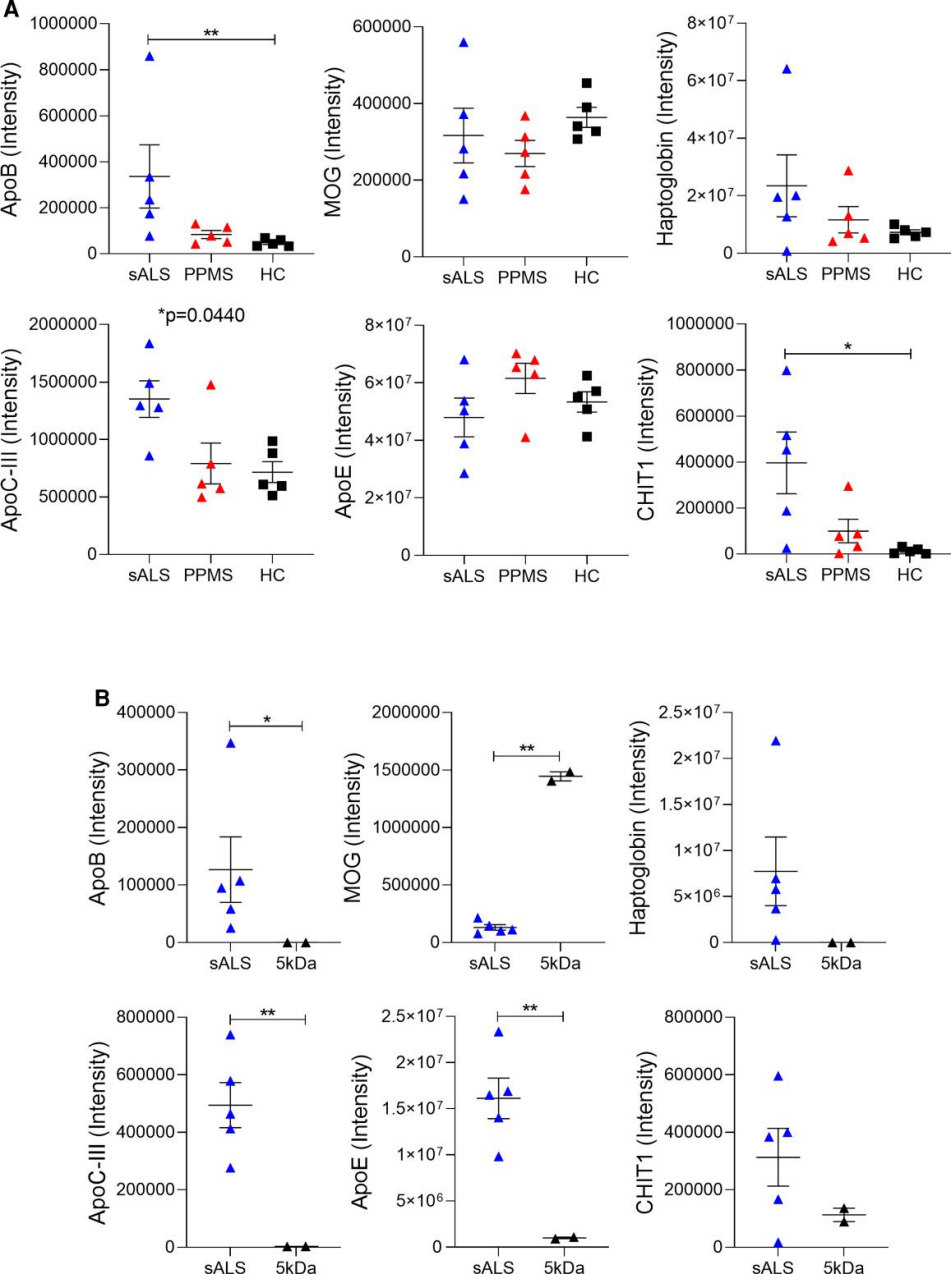
**Apolipoprotein B-100 is upregulated in sALS CSF and downregulated post-filtration.** (**A**) CSF protein intensities of apolipoprotein B-100 (ApoB), myelin oligodendrocyte glycoprotein (MOG), haptoglobin, apolipoprotein C-III (ApoC-III), apolipoprotein E (ApoE), and chitotriosidase1 (CHIT1) in patients with sALS (*n* = 5) or primary progressive multiple sclerosis (PPMS) (*n* = 5), and healthy individuals (HC) (*n* = 5), as determined by global proteome profiling using HRM™ MS. (**B**) CSF protein levels in sALS CSF pre- and post-filtration with a 5 kDa MWCO tangential flow hollow-fibre filter. Data plotted as mean ± SEM. Each point represents an individual subject (**A** and **B**). **P < 0.01, *P < 0.05.

By eliminating all proteins detected at similar or reduced levels in sALS CSF compared with HC CSF, and by application of the molecular weight screening, we identified apolipoprotein B-100 (ApoB) as a likely candidate neurotoxic protein. ApoB, a 550 kDa protein previously implicated as a risk and prognostic factor associated with ALS,^25–27^ was significantly upregulated in sALS CSF versus HC CSF, downregulated in 5 kDa-filtered sALS CSF and the sole candidate which met the molecular weight criteria (Fig. 8A and B). The elevated ApoB levels in sALS CSF compared with HC and multiple sclerosis CSF were validated by ELISA, and we additionally detected low ApoB levels in SOD1, C9 and TARDBP CSF (Supplementary Fig. 3A), consistent with the absence of motor deficits and reduced extent or lack of ALS-like pathology in C9 and TARDBP CSF-injected mice, and SOD1 CSF-injected mice, respectively. Furthermore, we confirmed a reduction in ApoB levels in the 5, 100 and 300 kDa sALS CSF filtrates, corresponding to attenuated neurotoxic capacity, whereas ApoB concentration in the neurotoxic 750 kDa filtrate remained similar to pre-filtration levels (Supplementary Fig. 3B).

### ApoB recapitulates motor disability and motor neuron death

**Figure 9:**
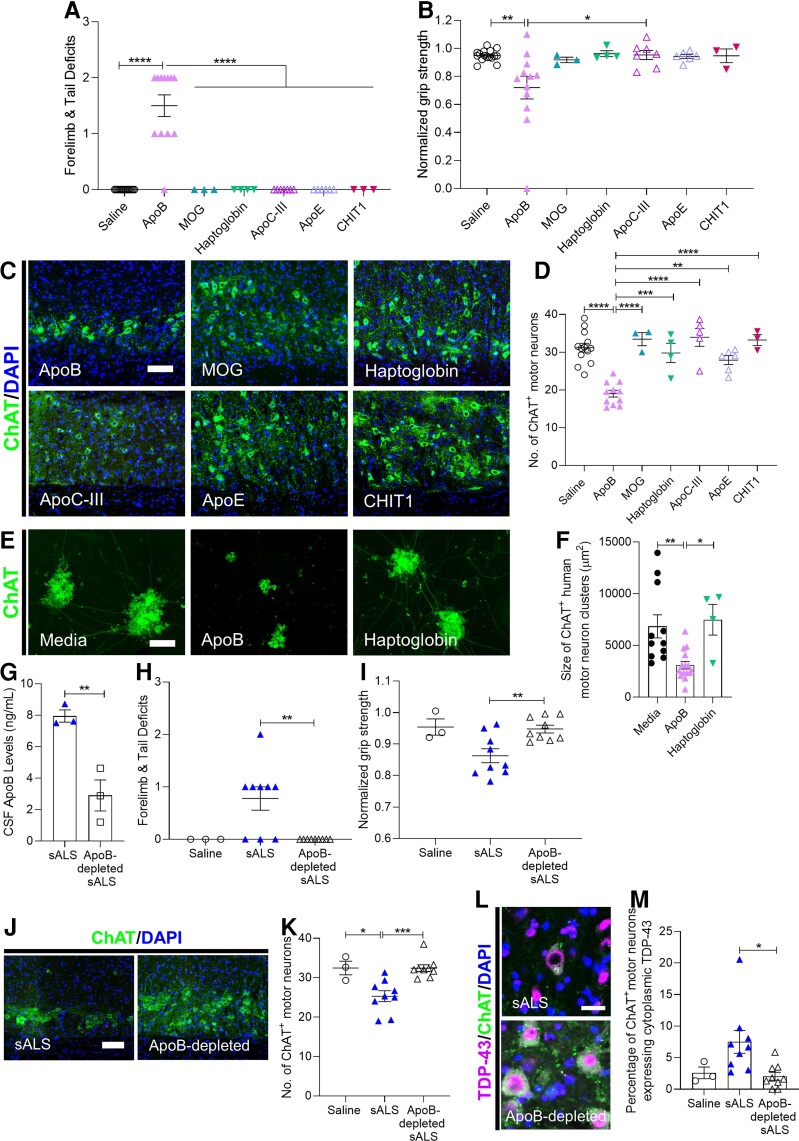
**Apolipoprotein B-100 induces motor neuron disability and motor neuron death.** (**A** and **B**) Motor deficit scores and normalized forelimb grip strength at 1 DPI of saline (*n* = 16 mice) or 1.5 µg/µL and 0.75 µg/uL: ApoB (*n* = 12 mice), MOG (*n* = 3 mice), haptoglobin (*n* = 4 mice), ApoC-III (*n* = 7 mice), ApoE (*n* = 6 mice), and CHIT1 (*n* = 3 mice). (**C**) Representative images of ChAT immunostaining in cervical spinal cords at 1 DPI of saline or 0.75 µg/µL ApoB, MOG, haptoglobin, ApoC-III, ApoE, and CHIT1. Scale bar, 100 µm. (**D**) Quantification of the number of ChAT^+^ motor neurons in cervical ventral horns at 1 DPI of saline (*n* = 13 mice) or 1.5 µg/µL and 0.75 µg/µL: ApoB (*n* = 11 mice), MOG (*n* = 3 mice), haptoglobin (*n* = 4 mice), ApoC-III (*n* = 5 mice), ApoE (*n* = 6 mice), and CHIT1 (*n* = 3 mice). (**E**) ChAT immunostaining of human iPSC-derived motor neurons cultured in motor neuron maintenance medium for 8 days, then treated for 24 h with 0.05 µg/µL ApoB or haptoglobin. Scale bar, 100 µm. (**F**) Quantification of the area of ChAT^+^ human motor neuron clusters 24 h following treatment with 0.05 µg/µL ApoB or haptoglobin. (**G**) ELISA measurements of ApoB levels in sALS CSF before and after ApoB immunodepletion. (**H** and **I**) Motor deficit scores and normalized forelimb grip strength at 1 DPI of saline (*n* = 3 mice), sALS CSF (*n* = 2; *n* = 9 mice), ApoB-depleted sALS CSF (*n* = 2; *n* = 9 mice). (**J**) Representative images of ChAT immunostaining in cervical spinal cords at 1 DPI of sALS CSF or ApoB-depleted sALS CSF. Scale bar, 100 µm. (**K**) Quantification of the number of ChAT^+^ motor neurons in cervical ventral horns at 1 DPI. Saline (*n* = 3 mice), sALS CSF (*n* = 9 mice), ApoB-depleted sALS CSF (*n* = 9 mice). (**L**) Representative images of TDP-43 immunostaining in ChAT^+^ motor neurons at 1 DPI of sALS CSF or ApoB-depleted sALS CSF. Scale bar, 25 µm. (**M**) Quantification of the number of ChAT^+^ motor neurons displaying TDP-43 exclusively in the cytoplasm at 1 DPI of saline (*n* = 3 mice), sALS CSF (*n* = 9 mice) or ApoB-depleted sALS CSF (*n* = 9 mice). Data plotted as mean ± SEM. Each point represents an individual mouse (**A**, **B**, **D**, **H**, **I**, **K**, and **M**), individual well (**F**), or CSF sample (**G**). One-way ANOVA with Bonferroni’s test or *t*-test. ****P < 0.0001, ***P < 0.001, **P < 0.01, *P < 0.05.

To verify whether ApoB could induce disability and degeneration of motor neurons, native human ApoB protein was delivered intrathecally in mice. We found that ApoB-injected mice displayed significant impairments in forelimb function, weaker grip strength, and ChAT^+^ motor neuron loss in the cervical spinal cord (Fig. 9A-D). ApoB had no impact on NeuN^+^ neurons in the spinal cord dorsal grey matter, olig2^+^ oligodendrocyte lineage cells or myelin integrity, suggesting that motor disability in ApoB-injected mice can be specifically attributed to motor neuron death (Supplementary Fig. 3C-F). Reactive astrogliosis and microglial activation were also triggered by ApoB, as indicated by higher GFAP expression and morphological changes in Iba1^+^ microglia; however NF-H and GLT-1 expression were unaffected suggesting that additional CSF factors may be contributing to these pathological changes observed in sALS CSF-injected mice (Supplementary Fig. 4). To determine the specificity of our ApoB findings, we tested other proteins from the proteomic analyses which were also significantly reduced in filtered sALS CSF or have been implicated in other neurodegenerative disorders. Myelin oligodendrocyte glycoprotein (MOG), haptoglobin, apolipoprotein C-III (ApoC-III), and apolipoprotein E (ApoE), all failed to recapitulate the motor disability and motor neuron degeneration observed with sALS CSF and ApoB (Fig. 9A-D). We also did not observe pathological changes with chitotriosidase (CHIT1), a protein recently implicated in ALS pathogenesis.^28^ These results are consistent with our CSF filtration findings, as all of these proteins are smaller than 300 kDa and are therefore present in the 300 kDa filtrate which had no effect upon intrathecal administration in mice. We further confirmed ApoB-induced cellular neurotoxicity in human iPSC-derived motor neurons, as indicated by significantly smaller ChAT^+^ cluster sizes following 24 h ApoB treatment but not with any control proteins tested (Fig. 9E and F; Supplementary Fig. 5A-C). In contrast, the same ApoB treatment conditions had no adverse effects on human primary astrocyte numbers (Supplementary Fig. 5D-F). Our data demonstrate that ApoB alone is sufficient to induce motor disability and specific degeneration of motor neurons, recapitulating the neurotoxic effects observed with sALS CSF injections.

### Targeted ApoB removal from sALS CSF attenuates neurotoxic capacity

To determine whether targeted removal of ApoB from sALS CSF would attenuate its neurotoxic capacity, ApoB immunodepletion was performed using ApoB antibody-coated Dynabeads® and reduction was confirmed by ELISA (Fig. 9G). In a new cohort of mice, we replicated the previously observed neurotoxic effects in sALS CSF-injected mice, while mice injected with ApoB-depleted sALS CSF from the same patients did not exhibit motor deficits, motor neuron loss or pathological TDP-43 translocation to the cytoplasm (Fig. 9H-M). However, other pathological changes were similar between mice injected with sALS CSF and ApoB-depleted CSF, suggesting that other components in sALS CSF also contribute to the induction of reactive astrogliosis, microglial activation, NF-H upregulation and excitotoxic changes (Supplementary Fig. 6A-E). Attenuation of sALS CSF neurotoxicity via ApoB depletion was also observed in vitro as human iPSC-derived motor neurons incubated with sALS CSF had significantly smaller clusters compared to the control media group, whereas motor neurons treated with ApoB-depleted sALS CSF were unaffected (Supplementary Fig. 6F and G). As a control, IgGs were depleted from sALS CSF using uncoated Dynabeads®. IgG depletion did not impact neurotoxicity as IgG-depleted sALS CSF was still able to induce motor deficits and motor neuron loss in mice, thereby excluding pathogenic antibodies as the neurotoxic candidate in sALS CSF (Supplementary Fig. 7). These studies further implicate ApoB as the primary neurotoxic component in sALS CSF contributing to neurodegeneration.

## Discussion

Here we present a novel CSF-mediated adult wild-type mouse model specific for the predominant sporadic form of ALS. Our study provides the first in vivo evidence demonstrating differences in neurotoxicity of CSF obtained from sALS patients, and fALS patients harbouring mutations in *SOD1, C9orf72* or *TARDBP*. Only sALS CSF consistently induced motor disability, upper and lower motor neuron degeneration, TDP-43 cytoplasmic translocation and other hallmark ALS-like pathology in mice. The moderate extent of neurotoxic effects induced by C9orf72 and TARDBP CSF, as well as the lack of effects induced by SOD1 CSF confirms the specificity of our sALS animal model. Combining sALS CSF filtration studies with global proteomic analyses led to the identification of ApoB as the candidate neurotoxic protein, which was validated both *in vivo* and *in vitro* with human ApoB protein alone recapitulating neurotoxic effects, and ApoB immunodepletion from sALS CSF mitigating neurotoxicity. Our data highlight fundamental differences in pathophysiological mechanisms underlying sporadic and familial forms of the disease, with genetic mutations perhaps increasing intrinsic vulnerability of motor neurons to stress in fALS, whereas neurotoxic levels of ApoB circulating in CSF contributes to neurological damage in sALS. This emphasizes the necessity of tailoring therapeutic approaches to specific ALS subtypes and the importance of resolving the toxic CSF environment in sALS. A previous *in vitro* study reported similar neurotoxic effects of CSF from sALS patients and patients with *SOD1* mutations on rat embryonic spinal cord cultures;^9^ however, apoptosis was measured in a mixed population of cells including neurons and glia, rather than motor neurons specifically. Future large-scale studies should be conducted to fully characterize the CSF profiles of patients with different ALS subtypes.

It has been previously reported that levels of CHIT1 are elevated in ALS CSF and that CHIT1 protein can induce motor neuron loss when injected into rat neonates.^28^ Although higher CHIT1 levels in sALS CSF were confirmed in our study, our filtration studies excluded CHIT1 as the neurotoxic candidate since both 100 and 300 kDa-filtered sALS CSF which retained the 51 kDa CHIT1 protein had no effect on motor function or motor neurons upon injection into the cervical subarachnoid space. Furthermore, CHIT1 protein also did not induce any neurotoxic effects in our animal model. This discrepancy in CHIT1 neurotoxic capacity might reflect differences in species and/or age, with rat neonates perhaps being more vulnerable to neurotoxicity than the adult mice used in our experiments.

Other studies reporting neurotoxic effects of ALS CSF in vivo have delivered much larger volumes of CSF (100 µL) into the lateral ventricles of adult rats^13^ or TDP-43 transgenic mice.^14^ An advantage of our cervical subarachnoid space injections is that only a single injection of 3 µL sALS CSF is sufficient to induce persistent motor disability and widespread neurotoxic effects, including pathological TDP-43 translocation, which was previously only observed in TDP-43 transgenic mice but not wild-type mice.^14^ The extent of disability and cell death is the same at 1 and 28 DPI, indicating the occurrence of acute irreparable neurological injury rather than progressive degeneration in our model. Increasing levels of ApoB in blood associated with a higher risk of developing ALS have been reported during the decade prior to ALS diagnosis;^25,27^ however, it remains unknown what causes the increase in CSF ApoB and whether a spike in CSF ApoB levels triggers motor neuron degeneration or whether elevated ApoB is a downstream consequence exacerbating disease pathophysiology in sALS.

A caveat of our CSF-mediated sALS model is the assumption that neurotoxicity arises from an overabundance rather than a depletion of a CSF component. Our work does not exclude the possibility that depleted levels of a protein could also lead to dysregulation of pertinent cell survival pathways, for instance, or the possibility that other pathological mechanisms contributing to motor neuron degeneration can occur simultaneously with ApoB-induced cell death. Prior studies have demonstrated that lipid-free ApoB has cytotoxic effects on macrophages, CHO cells and HepG2 cells via plasma membrane disruption, increased calcium influx and caspase-3 activation.^29^ We also observed evidence of activated caspase-3 in motor neurons of sALS CSF-injected mice; however, future studies are needed to determine the exact mechanisms and cell death signalling pathways by which elevated levels of ApoB in CSF trigger motor neuron toxicity and disease progression in sALS. Potential mechanisms of neurotoxicity could involve cell death signalling pathways triggered by ApoB binding to its receptors such as the low-density lipoprotein receptor (LDLR)^30^ and/or sortilin receptor.^31^ For instance, it has been reported that inactivation of liver X receptor (LXR) can lead to degeneration of motor neurons in mice,^32^ and it is possible that increased CSF ApoB levels could modulate the LXR-inducible degrader of the LDLR (IDOL)-LDLR pathway in a detrimental manner.^33^ Additionally, sortilin activation is known to involve signalling pathways which eventually lead to caspase activation and cell death,^34^ and it is possible that sALS CSF-induced motor neuron degeneration is mediated by an ApoB/sortilin-mediated cell death signalling pathway.

This study provides a novel CSF ApoB-mediated mechanism of motor neuron degeneration which appears to occur in the most common sporadic form of ALS, but not fALS arising due to mutations in *SOD1*, *C9orf72* or *TARDBP*. Our data clearly show that ApoB is sufficient to induce motor disability and motor neuron degeneration, and its removal from sALS CSF via filtration or immunodepletion attenuates neurotoxicity, establishing a novel sALS-specific therapeutic target and proof-of-concept to support targeted reduction of ApoB via CSF pheresis^35–37^ as a therapeutic strategy for sALS patients.

## Supplementary Material

fcac207_Supplementary_Data

## Abbreviations

ApoB =: apolipoprotein B-100
DCs =: disease controls
DPI =: day(s) post-injection
fALS =: familial amyotrophic lateral sclerosis
HCs =: healthy controls
sALS =: sporadic amyotrophic lateral sclerosis
