# Supplementary material for: Apolipoprotein B-100-mediated motor neuron degeneration in sporadic amyotrophic lateral sclerosis
Source: Brain Commun. 2022 Aug 22;4(4):fcac207. doi: 10.1093/braincomms/fcac207 (PMC9416068; doi:10.1093/braincomms/fcac207)
Supplement: fcac207_Supplementary_Data [file fcac207_supplementary_data.pdf]

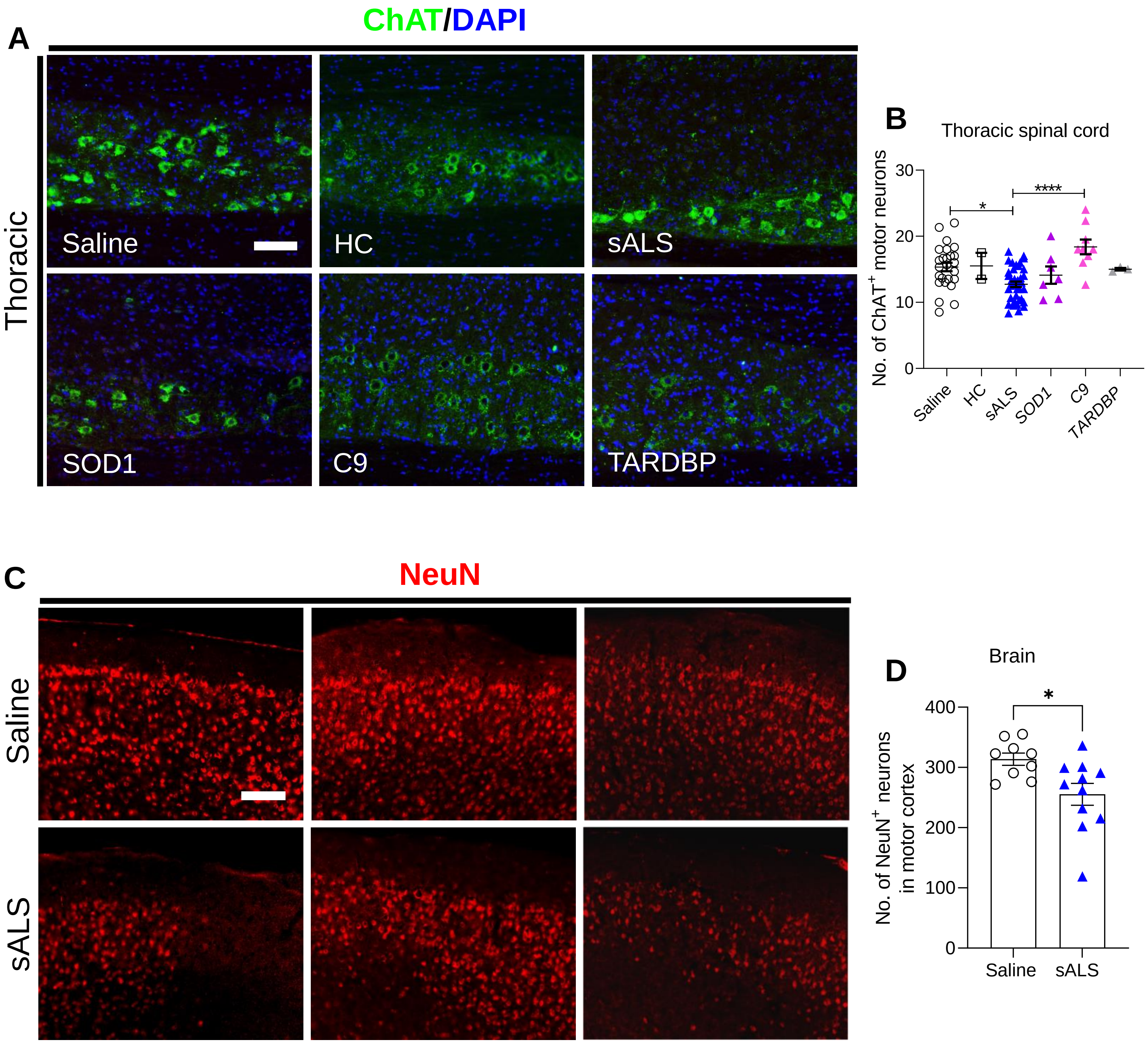


**Supplementary Figure 1. Motor neuron loss extends to thoracic spinal cord and upper motor neurons in the motor cortex at 1 day following intrathecal delivery of sALS CSF.** (**A**) Representative images of thoracic spinal cords immunostained for ChAT at 1 day post injection (DPI) of saline, healthy control (HC) CSF, CSF from patients with sALS, *SOD1* ALS, *C9* ALS, or *TARDBP* ALS. Scale bar, 100 µm. (**B**) Quantification of the number of ChAT$^+$ motor neurons in thoracic ventral horns at 1 DPI. Saline ($n$ = 25 mice), HC ($n$ = 2 mice), sALS ($n$ = 38 mice), SOD1 ($n$ = 7 mice), C9 ($n$ = 9 mice), TARDBP ($n$ = 3 mice). (**C**) Representative images of NeuN immunostaining in the motor cortex at 1 DPI from 3 different mice per group. Scale bar, 100 µm. (**D**) Quantification of the number of NeuN$^+$ neurons in the motor cortex at 1 DPI of saline ($n$ = 9 mice) or sALS CSF ($n$ = 11 mice).

Data plotted as mean ± s.e.m. Each point represents an individual mouse (**B** and **D**). One-way ANOVA with Bonferroni's test (**B**) and t-test (**D**). ****$P$ < 0.0001, *$P$ < 0.05.

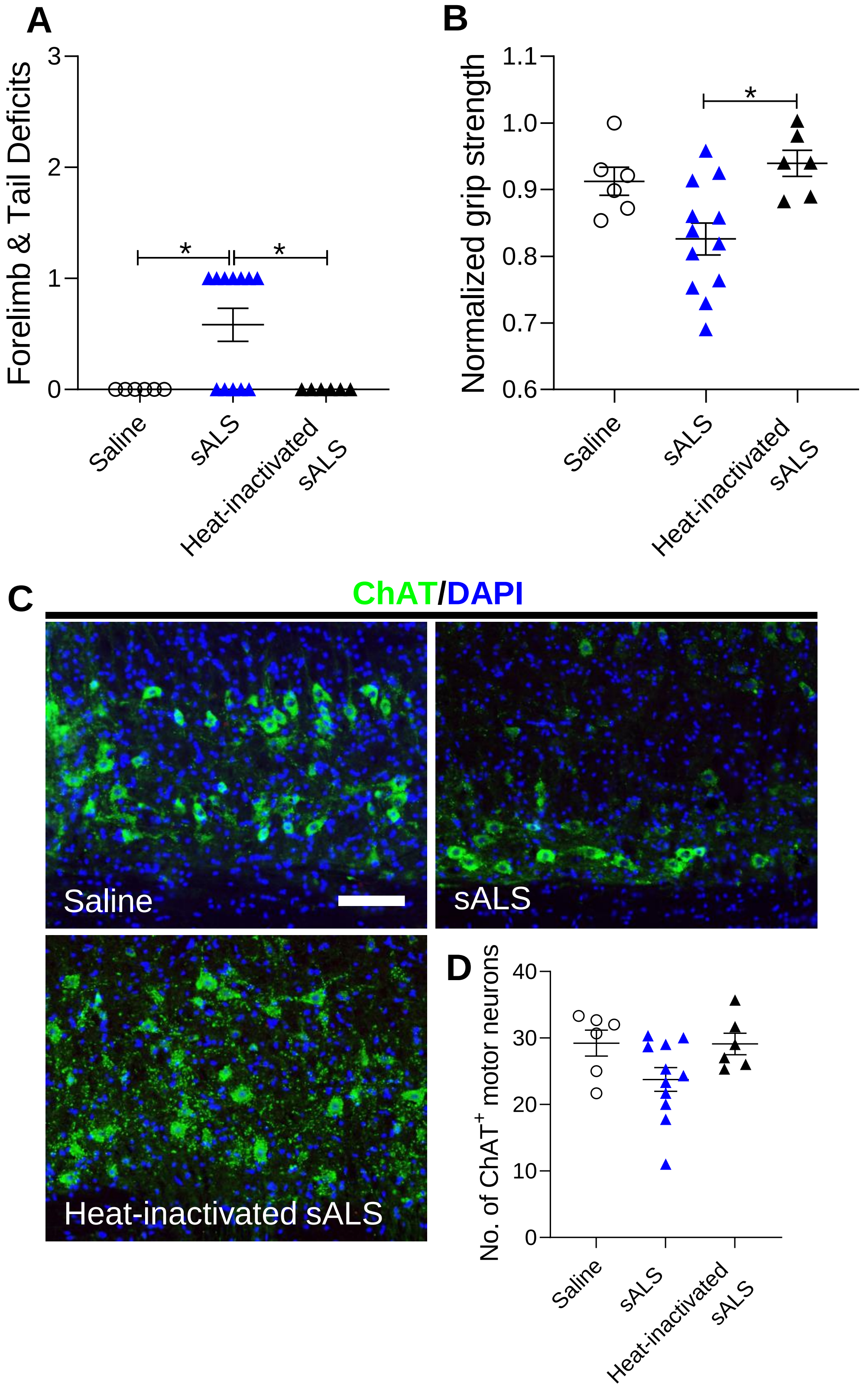


**Supplementary Figure 2. Heat inactivation attenuates neurotoxicity of sALS CSF.** (**A** and **B**) Motor deficit scores and normalized forelimb grip strength at 1 day post injection (DPI) of saline (*n* = 6 mice), sALS CSF (*n* = 12 mice), or sALS CSF heated to 80$^{o}$C for 1 hour (*n* = 6 mice). (**C**) Representative images of ChAT immunostaining in cervical spinal cords at 1 DPI. Scale bar, 100 µm. (**D**) Quantification of the number of ChAT$^{+}$ motor neurons in cervical ventral horns at 1 DPI. Saline (*n* = 6 mice), sALS CSF (*n* = 11 mice), heat-inactivated sALS CSF (*n* = 6 mice).

Data plotted as mean ± s.e.m. Each point represents an individual mouse (**A**, **B** and **D**). One-way ANOVA with Bonferroni's test. *$P < 0.05$.

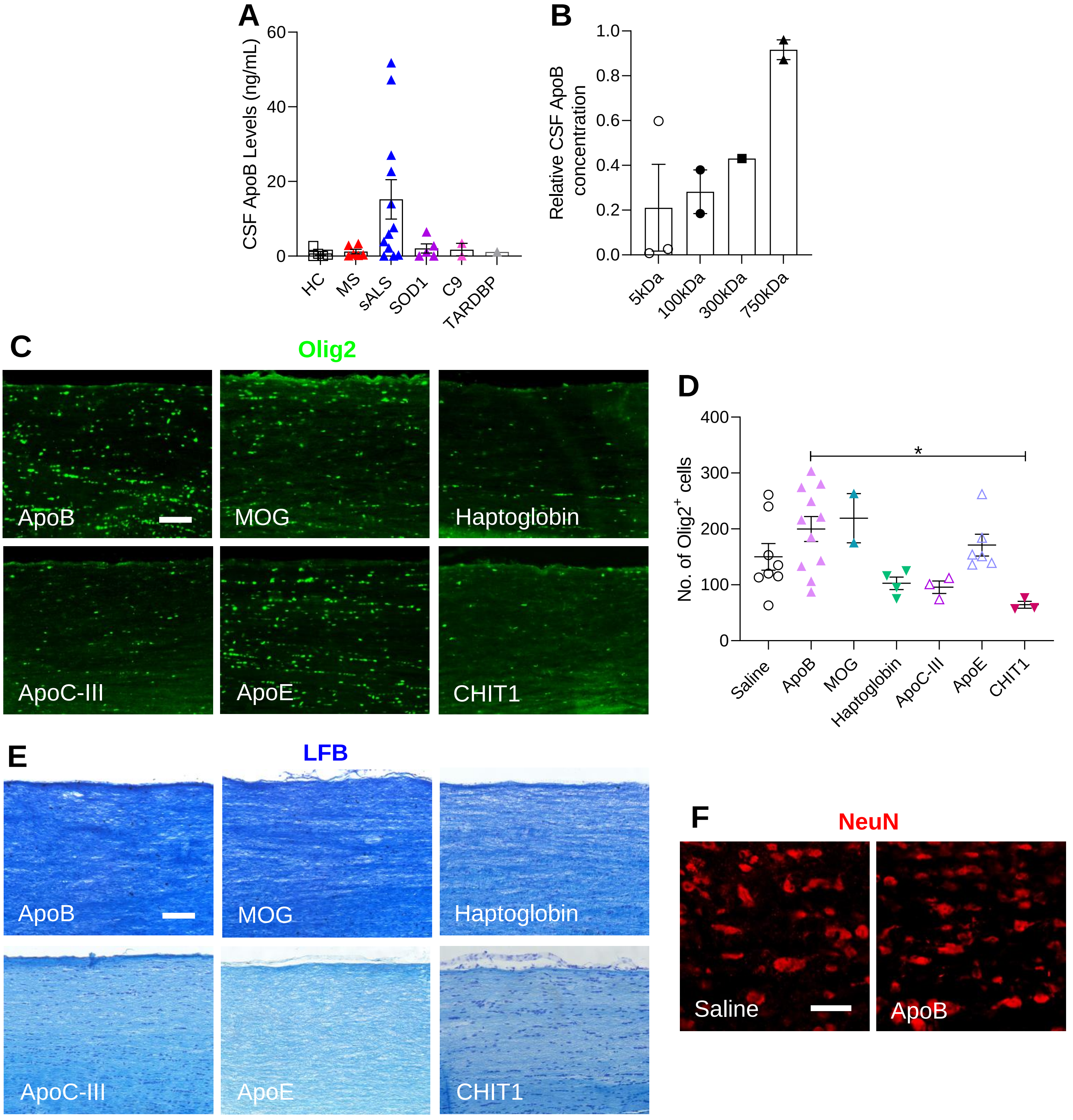


**Supplementary Figure 3. ApoB toxicity does not affect oligodendrocytes, myelin, or dorsal grey matter NeuN$^+$ neurons.** (**A**) ELISA measurements of CSF ApoB protein levels in a different cohort of samples: healthy control (HC) CSF (*n* = 5), MS CSF (*n* = 6), sALS CSF (*n* = 12), SOD1 CSF (*n* = 5), C9 CSF (*n* = 2), TARDBP CSF (*n* = 1), and (**B**) following CSF filtration. (**C**) Representative images of Olig2 immunostaining in cervical spinal cords at 1 DPI. Scale bar, 100 µm. (**D**) Quantification of the number of Olig2$^+$ oligodendrocytes in the dorsal white matter at 1 DPI of saline (*n* = 8 mice) or 1.5 µg/µl and 0.75 µg/ul: ApoB (*n* = 11 mice), MOG (*n* = 2 mice), haptoglobin (*n* = 4 mice), ApoC-III (*n* = 3 mice), ApoE (*n* = 6 mice), and CHIT1 (*n* = 3 mice). (**E**) Representative images of luxol fast blue (LFB) staining for myelin in dorsal white matter. Scale bar, 100 µm. (**F**) Representative images of NeuN immunostaining in the dorsal grey matter of cervical spinal cords. Scale bar, 50 µm.

Data plotted as mean ± s.e.m. Each point represents an individual mouse (**D**). One-way ANOVA with Bonferroni's test. *$P < 0.05$.

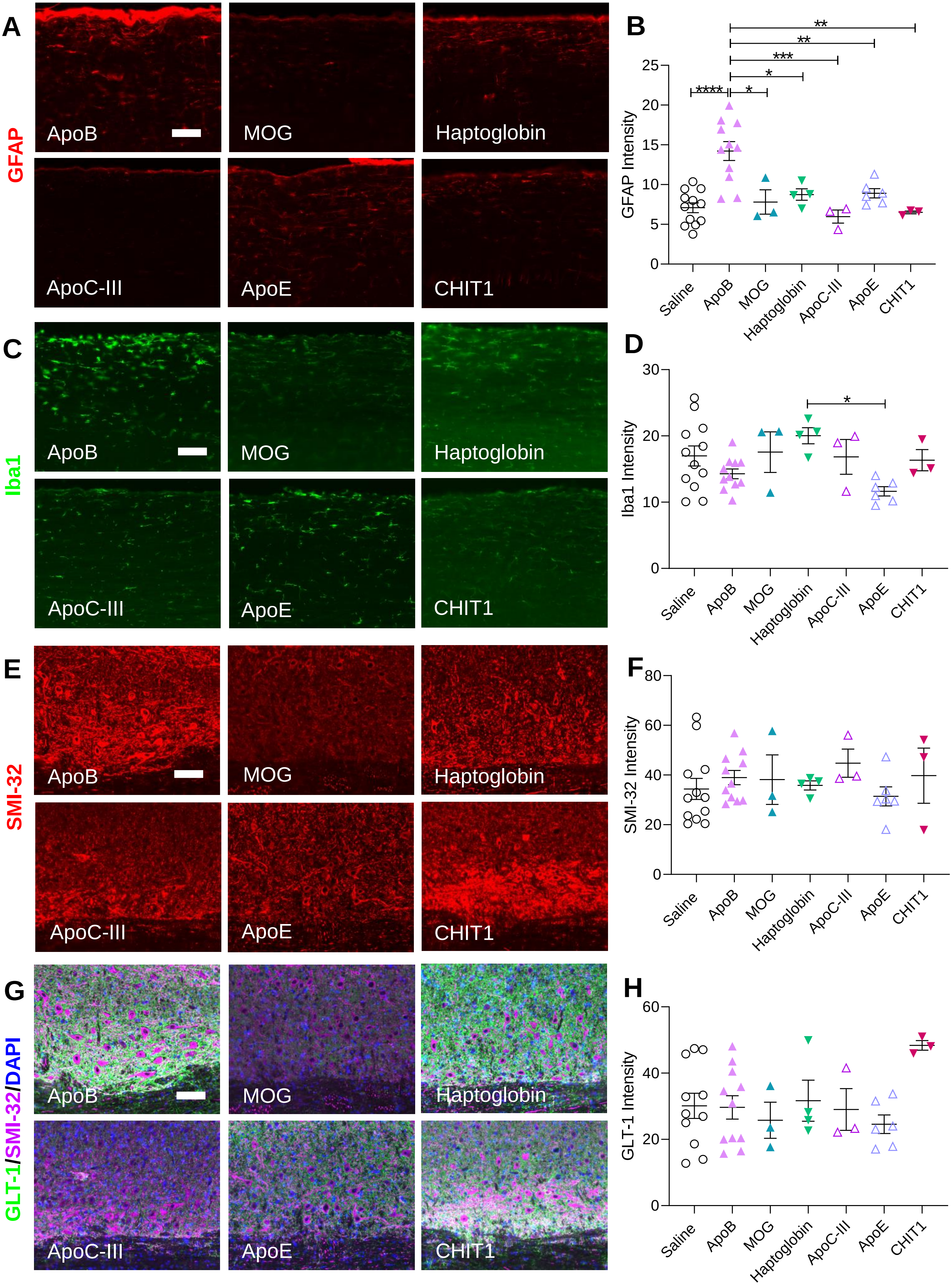
A
GFAP
ApoB
MOG
Haptoglobin
ApoC-III
ApoE
CHIT1
B
GFAP Intensity
0
5
10
15
20
25
****
*
*
***
**
**
Saline
ApoB
MOG
Haptoglobin
ApoC-III
ApoE
CHIT1
C
Iba1
ApoB
MOG
Haptoglobin
ApoC-III
ApoE
CHIT1
D
Iba1 Intensity
0
10
20
30
*
Saline
ApoB
MOG
Haptoglobin
ApoC-III
ApoE
CHIT1
E
SMI-32
ApoB
MOG
Haptoglobin
ApoC-III
ApoE
CHIT1
F
SMI-32 Intensity
0
20
40
60
80
Saline
ApoB
MOG
Haptoglobin
ApoC-III
ApoE
CHIT1
G
GLT-1/SMI-32/DAPI
ApoB
MOG
Haptoglobin
ApoC-III
ApoE
CHIT1
H
GLT-1 Intensity
0
20
40
60
Saline
ApoB
MOG
Haptoglobin
ApoC-III
ApoE
CHIT1

**Supplementary Figure 4. ApoB induces astrocyte and microglial activation.** (**A**, **C**, **E** and **G**) Representative images of GFAP (**A**), Iba1 (**C**), SMI-32 (**E**), and GLT-1 (**G**) immunostaining in cervical spinal cords at 1 DPI. Scale bar, 100 µm. (**B**, **D**, **F** and **H**) Quantification of immunostaining intensities in the dorsal white matter (**B** and **D**), areas surrounding motor neurons (**F**) and ventral grey matter (**H**) at 1 DPI of saline ($n$ = 12), ApoB ($n$ = 11), MOG ($n$ = 3), haptoglobin ($n$ = 4), ApoC-III ($n$ = 3), ApoE ($n$ = 6), or CHIT1 ($n$ = 3).
Data plotted as mean ± s.e.m. Each point represents an individual mouse (**B**, **D**, **F** and **H**). One-way ANOVA with Bonferroni's test. ****$P$ < 0.0001, ***$P$ < 0.001, **$P$ < 0.01, *$P$ < 0.05.

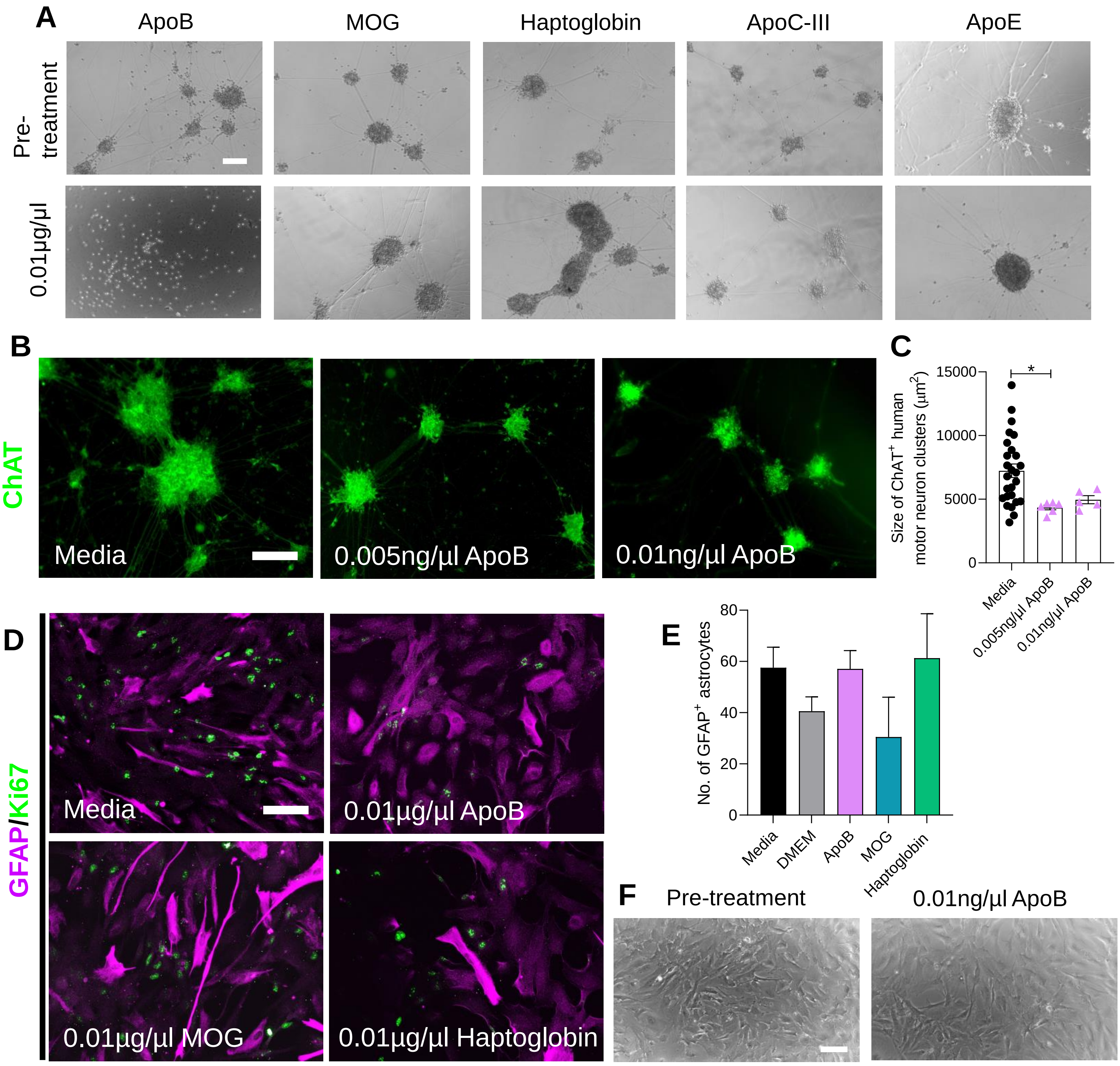


**Supplementary Figure 5. Human motor neurons are more vulnerable to ApoB-induced cytotoxicity than human astrocytes.** (**A**) Bright field images of human iPSC-derived motor neurons cultured in motor neuron maintenance medium at 8 days in vitro (DIV), and at 9 DIV, 24 hours post-treatment with 0.01 µg/µl ApoB, MOG, haptoglobin, ApoC-III or ApoE diluted in medium. Scale bar, 100 µm. (**B**) Representative images of ChAT immunocytochemistry on human iPSC-derived motor neurons cultured in motor neuron maintenance medium for 8 days then incubated with 0.005 ng/µl or 0.01 ng/µl ApoB for 24 hours. Scale bar, 100 µm. (**C**) Area of $ChAT^{+}$ human motor neuron clusters 24 hours following treatment with ApoB. (**D**) Representative images of human primary astrocytes cultured in astrocyte growth medium for 5 days then incubated for 24 hours with 0.01 µg/µl ApoB, MOG, or haptoglobin diluted in DMEM. Scale bar, 100 µm. (**E**) Quantification of the number of $GFAP^{+}$ human astrocytes following 24-hour treatment with 0.01 µg/µl proteins. (**F**) Bright field images of human primary astrocytes pre- and post-24-hour incubation with 0.01 ng/µl ApoB. Scale bar, 100 µm. Data plotted as mean ± s.e.m. Each point represents an individual well (**C**). One-way ANOVA with Bonferroni's test. $*P < 0.05$.

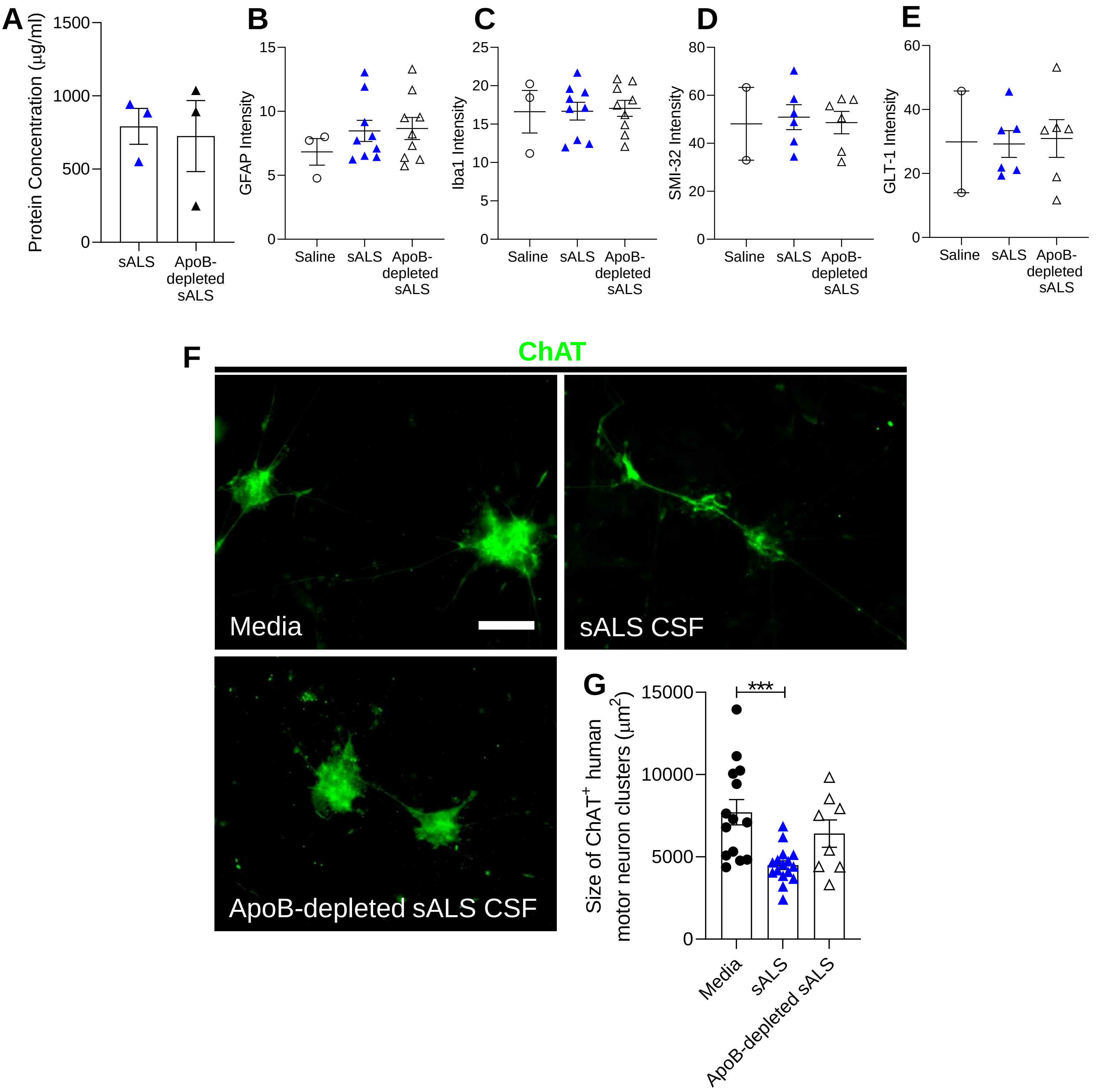


**Supplementary Figure 6. ApoB-depletion attenuates sALS CSF-induced human motor neuron death.** (**A**) Protein concentration in sALS CSF ($n$ = 3) and ApoB-depleted sALS CSF ($n$ = 3). (**B-E**) Quantification of intensities of GFAP (**B**), Iba1 (**C**), SMI-32 (**D**), and GLT-1 (**E**) immunostaining in cervical spinal cords at 1 day post injection of saline, sALS CSF, or ApoB-depleted sALS CSF in mice. (**F**) Representative images of ChAT immunocytochemistry on human iPSC-derived motor neurons cultured in motor neuron maintenance medium for 8 days then incubated with 50% sALS CSF ($n$ = 3 patients) or ApoB-depleted sALS CSF for 24 hours. Scale bar, 100 μm. (**G**) Area of $ChAT^+$ human motor neuron clusters 24 hours following treatment with 50% sALS CSF or ApoB-depleted sALS CSF.

Data plotted as mean ± s.e.m. Each point represents an individual mouse (**B-E**) or individual wells (**G**). One-way ANOVA with Bonferroni's test. ***$P$ < 0.001.

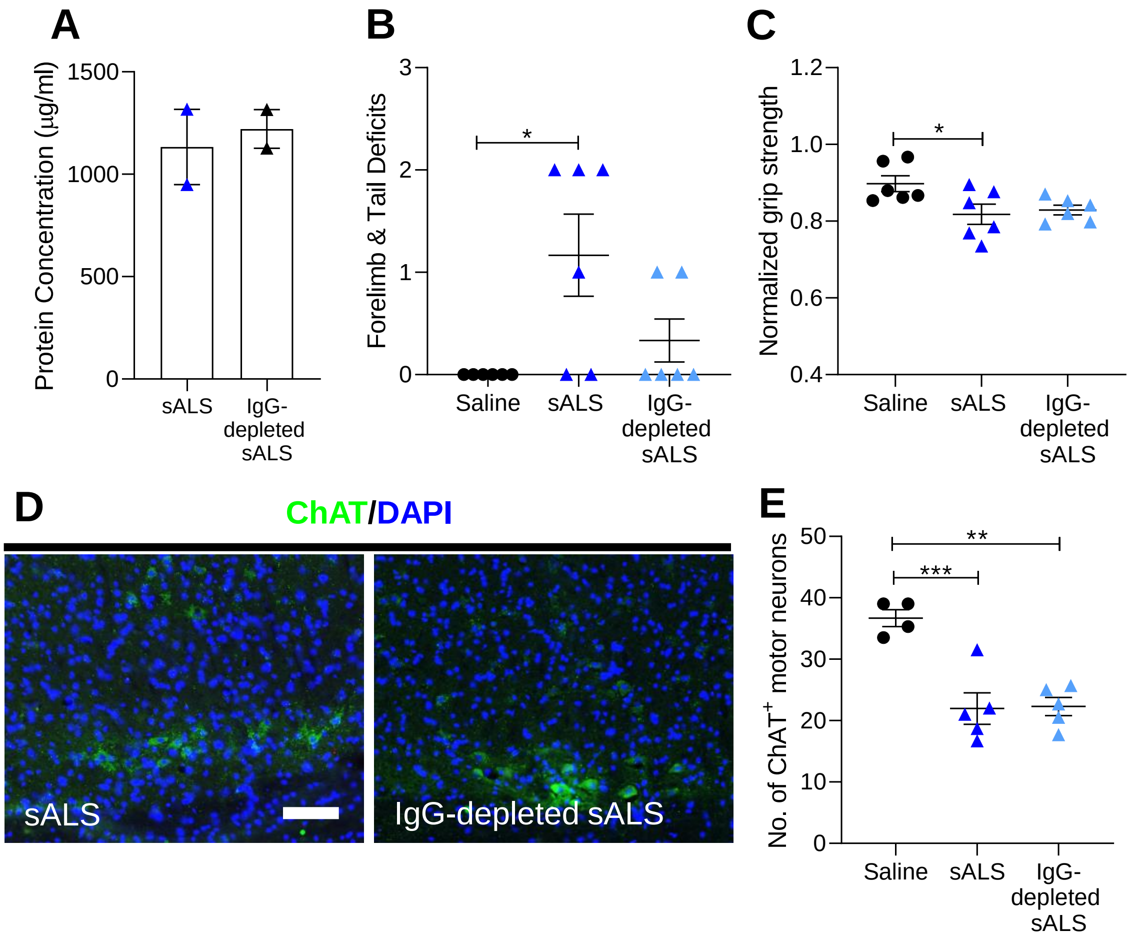


**Supplementary Figure 7. IgG-depleted sALS CSF maintains neurotoxic capacity.** (**A**) Protein concentration in sALS CSF (*n* = 2) and IgG-depleted sALS CSF (*n* = 2). (**B** and **C**) Motor deficit scores and normalized forelimb grip strength at 1 day post injection of saline (*n* = 6 mice), sALS CSF (*n* = 6 mice), or IgG-depleted sALS CSF (*n* = 6 mice). (**D**) Representative images of ChAT immunostaining in cervical spinal cords at 1 DPI. Scale bar, 100 µm. (**E**) Quantification of the number of $ChAT^+$ motor neurons in cervical ventral horns at 1 DPI. Saline (*n* = 4 mice), sALS CSF (*n* = 5 mice), IgG-depleted sALS CSF (*n* = 5 mice).

Data plotted as mean ± s.e.m. Each point represents an individual mouse (**B**, **C** and **E**). One-way ANOVA with Bonferroni's test. ***$P < 0.001$, **$P < 0.01$, *$P < 0.05$.
